# Epigenetic repression of miR-17 contributed to di(2-ethylhexyl) phthalate-triggered insulin resistance by targeting Keap1-Nrf2/miR-200a axis in skeletal muscle

**DOI:** 10.7150/thno.45253

**Published:** 2020-07-23

**Authors:** Jie Wei, Qiongyu Hao, Chengkun Chen, Juan Li, Xikui Han, Zhao Lei, Tao Wang, Yinan Wang, Xiang You, Xiaoxuan Chen, Huasheng Li, Yuxin Ding, Weihao Huang, Yangyang Hu, Shuirong Lin, Heqing Shen, Yi Lin

**Affiliations:** 1 Department of Basic Medical Sciences, School of Medicine, Xiamen University, Xiamen 361102, China.; 2 State Key Laboratory of Molecular Vaccinology and Molecular Diagnostics, School of Public Health, Xiamen University, Xiamen 361102, China.; 3 Division of Cancer Research and Training, Department of Internal Medicine, Charles R. Drew University of Medicine and Science, David Geffen UCLA School of Medicine, and UCLA Jonsson Comprehensive Cancer Center, 1748 E. 118th Street, Los Angeles, CA, 90059, USA.; 4 The First Affiliated Hospital of Xiamen University, Xiamen, 361003, China.

**Keywords:** Environmental endocrine-disrupting chemical, Insulin resistance, microRNA, Oxidative stress, Skeletal muscle

## Abstract

**Rationale:** Skeletal muscle insulin resistance is detectable before type 2 diabetes is diagnosed. Exposure to di(2-ethylhexyl) phthalate (DEHP), a typical environmental endocrine-disrupting chemical, is a novel risk factor for insulin resistance and type 2 diabetes. This study aimed to explore insulin signaling regulatory pathway in skeletal muscle of the DEHP-induced insulin-resistant mice and to investigate potential therapeutic strategies for treating insulin resistance.

**Methods:** C57BL/6J male mice were exposed to 2 mg/kg/day DEHP for 15 weeks. Whole-body glucose homeostasis, oxidative stress and deregulated miRNA-mediated molecular transduction in skeletal muscle were examined. microRNA (miRNA) interventions based on lentiviruses and adeno-associated viruses 9 (AAV9) were performed.

**Results:** Dnmt3a-dependent promoter methylation and lncRNA Malat1-related sponge functions cooperatively downregulated miR-17 in DEHP-exposed skeletal muscle cells. DEHP suppressed miR-17 to disrupt the Keap1-Nrf2 redox system and to activate oxidative stress-responsive Txnip in skeletal muscle. Oxidative stress upregulated miR-200a, which directly targets the 3'UTR of *Insr* and *Irs1*, leading to hindered insulin signaling and impaired insulin-dependent glucose uptake in skeletal muscle, ultimately promoting the development of insulin resistance. AAV9-induced overexpression of miR-17 and lentivirus-mediated silencing of miR-200a in skeletal muscle ameliorated whole-body insulin resistance in DEHP-exposed mice.

**Conclusions:** The miR-17/Keap1-Nrf2/miR-200a axis contributed to DEHP-induced insulin resistance. miR-17 is a positive regulator, whereas miR-200a is a negative regulator of insulin signaling in skeletal muscle, and both miRNAs have the potential to become therapeutic targets for preventing and treating insulin resistance or type 2 diabetes.

## Introduction

Type 2 diabetes (T2D) is a chronic disease characterized by hyperglycemia resulting from peripheral insulin resistance (IR) and pancreatic β cells failure. During the last decade, exposure to environmental endocrine-disrupting chemicals (EDCs) has been implicated as a novel contributor to the epidemic of T2D 1, 2. Di(2-ethylhexyl) phthalate (DEHP) is one of the most common EDCs used in consumer, medical, and construction products. Several cross-sectional analyses consistently showed that urinary concentrations of DEHP and its metabolites were associated with increased IR or T2D in US adolescents 3, 4, as well as adult men 5 and women 6. Another study focusing on the Canadian population, including males and females, and young and old individuals, also noted that exposure to DEHP might contribute to prediabetes 2. Consistent with epidemiological studies, the role of DEHP in glucometabolic disorders was shown in our previous studies *in vivo* and *in vitro* 7, 8, and further investigation is needed to elucidate the mechanism by which DEHP acts to induce this risk.

Skeletal muscle IR (SkM-IR) is an early event and primary deficiency in the process of T2D 9-11. Gestational exposure to DEHP predisposed offspring to glucometabolic dysfunction in adulthood by downregulating insulin signaling genes, including *Insr*, *Irs1*, and *Akt*, and inducing epigenetic alteration of* Glut4* in the gastrocnemius muscle of rats 12. The direct effects of DEHP on insulin signal transduction and Glut4 translocation were also confirmed in rat L6 myotubes 13. Interestingly, the antioxidants vitamins C and E alleviated DEHP-induced SkM-IR in rats 14. The role of oxidative stress in DEHP-induced IR was also emphasized in a cross-sectional pilot study in which exposure to DEHP at the community level promoted IR in 10-13-year-old children by inducing systemic oxidant stress, characterized by the increased urinary level of F2-isoprostane 15. Nrf2 is a master regulator of the cytoprotective program against oxidative stress and, more importantly, has the capability to detoxify DEHP 16, 17. Our previous study indicated a critical role for the Nrf2-mediated antioxidant response in DEHP-induced rat insulinoma INS-1 cells dysfunction 8. Whether DEHP-induced IR was also related to an impairment of the Nrf2 redox system in SkM is worthy of further study.

microRNAs (miRNAs) act as epigenetic regulators by posttranscriptionally repressing target mRNAs. We previously showed that miR-200a/141 acted to target Keap1 directly and then regulated Nrf2 under high-glucose conditions, resulting in diabetic nephropathy in mice 18. The function of the miR-200 family in regulating oxidative stress 19-21 and glucose homeostasis 22-25 has been demonstrated. In addition to miR-200a, our previous studies suggested the role of specific miRNA (including miR-338, miR-192 and miR-26a) modifications in regulating environmental cues such as bisphenol A and ambient particulate matter-induced disorders of glucose and lipid metabolism 26-29. To date, few studies regarding the influence of miRNA deregulation on DEHP-associated injury have been published. Therefore, this study intended to examine the mechanism by which the mutual functional status of the keap1-Nrf2 pathway and miRNAs, including miR-200a, contributed to DEHP-induced SkM-IR and, more importantly, to investigate potential targets to intervene in IR.

## Methods

### Animal Experiments

All animal experiments were carried out in accordance with the guidelines of the Xiamen University Institutional Committee for the Care and Use of Laboratory Animals (XMULAC20150081).

Three-week-old male healthy C57BL/6 mice were purchased from the SLAC Laboratory Animal Center (Shanghai, China) and housed (5 mice/cage) under specific pathogen-free conditions (Xiamen University Laboratory Animal Center, Xiamen, China) with controlled room temperature (22 ± 2 °C), humidity (55 ± 5%) and a 12:12 h light-dark cycle. Mice had ad libitum access to food and water. The diet (standard rodent chow diet, Xietong Organism Institute, Nanjing, China) contained 12% fat, 20.6% protein and 67.4% carbohydrates, with energy of 3.616 kJ/g. After 1 week of adaption, the mice were administered corn oil (Sigma-Aldrich, MO, USA) or 2 mg/kg/day of DEHP (J&K Chemical, Shanghai, China) dissolved in corn oil (Millipore-Sigma, MO, USA) by oral gavage. After 15 weeks of DEHP administration, the C57BL/6 mice were anaesthetized and sacrificed by decollation.

For antioxidant treatment, 2 mM N-acetylcysteine (NAC, Millipore-Sigma) was administered to DEHP-exposed mice in drinking water throughout the experimental period. For miR-200a inhibition, DEHP-exposed mice were administered anti-miR-200a lentivirus (SBO Medical Biotechnology, Shanghai, China) through intramuscular injections on the 1^st^, 5^th^, 10^th^, 15^th^ and 20th day for a total of five injections at a concentration of 1×10^7^ transducing units each time. For miR-17 overexpression in SkM, DEHP-exposed mice received adeno-associated virus 9 (AAV9)-delivered miR-17 (SBO Medical Biotechnology) at a titer of 5×10^9^ particles via intramuscular injections. The AAV9 vectors were delivered 4 weeks prior to SkM tissue collection.

### Cell culture and treatment

Mouse C2C12 myoblast cells (Shanghai Institute of Cell Biology, Chinese Academy of Sciences) were cultured in DMEM (Thermo Fisher Scientific, MA, USA) supplemented with 10% FBS (Thermo Fisher Scientific). When the C2C12 myoblasts reached 80% confluence, the cells were switched to differentiation medium consisting of DMEM supplemented with 2% horse serum (Thermo Fisher Scientific). Myotubes were used for experiments after 6 days of differentiation (Figure S3). Differentiated C2C12 myotubes were treated with serial concentrations (0, 1, 5, and 25 µM) of DEHP for 48 h.

For inhibition and overexpression of miR-200a and miR-17, C2C12 myotubes were transfected with 50 nM agomiR-200a or agomiR-17 (Ribo Bio, Guangzhou, China), 200 nM antagomiR-200a or antagomiR-17, and their corresponding controls for 48 h. To inhibit Txnip and Long noncoding RNA metastasis-associated lung adenocarcinoma transcript 1(lncRNA Malat1), C2C12 myotubes were transfected with the corresponding siRNAs at a final concentration of 100 nM (Santa Cruz Biotechnology, CA, USA). For the overexpression of Irs1, C2C12 myotubes were transfected with pEGFP-N1 and pEGFP-N1-Irs1 (Sangon Biotech, Shanghai, China) using Lipofectamine 3000 reagent (Life Technologies, CA, USA). For Nrf2 activation, C2C12 myotubes were pretreated with the chemical Nrf2 inducer sulforaphane (SFN, 5 μM, Millipore-Sigma) before DEHP exposure. 5-aza-2′-deoxycytidine (5-Aza, 10 μM, Millipore-Sigma) was applied to DEHP-exposed C2C12 myotubes to demonstrate the association of miR-17 downregulation with hypermethylation.

### Luciferase reporter assays

Wild-type and mutated mouse* Insr*, *Irs1* and *Keap1* 3′UTR sequences were PCR-amplified from cDNA using specific primers (**Table S1**). The wild-type (WT) or mutant (MT) 3′UTRs were cloned into the pmiR-RB-REPORT vector (Ribo Bio). For luciferase reporter assays, C2C12 myoblasts were cotransfected with the indicated 3′UTR luciferase reporter vectors and agomiR-200a, antagomiR-200a, agomiR-17, or antagomiR-17 for 48 h. The relative luciferase activities were measured using a dual-luciferase reporter assay kit (Promega, WI, USA).

### Metabolic measurements

For the glucose tolerance test (GTT), overnight-fasted mice were injected intraperitoneally with glucose at a dose of 2.0 g/kg. For the insulin tolerance test (ITT), 6-h-fasted mice were injected intraperitoneally with 0.75 U/kg insulin (Novolin R; Novo Nordisk AIS, Bagsvaerd, Denmark). Glucose values were converted into natural logarithm (Ln) and the slope of the decline in the blood glucose concentration from 0 to 30 min was calculated using linear regression [time × Ln (glucose)] and multiplied by 100 to obtain the constant rate for glucose disappearance (KITT) 30, 31. Blood samples were collected at regular intervals (0-120 min). Blood glucose was measured by a handheld glucose meter (Accu-Chek Active; Roche, Mannheim, Germany). Serum insulin was analyzed with ELISA kits (Alpco Diagnostics, NH, USA). For insulin signaling experiments, mice were fasted overnight and intraperitoneally injected with 1 U/kg insulin solution (Novolin R). After 10 min, the mice were sacrificed and gastrocnemius SkM were harvested. For the *in vitro* cultured model, the treated C2C12 myotubes were stimulated with 100 nM insulin (ProSpec-Tany Technogene, Ness Ziona, Israel) for 15 min before harvesting.

### Glucose uptake assay

2-Deoxyglucose (2-DG) uptake was assessed using a glucose uptake kit (ab136955, Abcam, Cambridge, UK). Briefly, C2C12 myotubes were starved in serum-free medium overnight and incubated for 40 min at 37 °C in the absence or presence of 100 nM insulin in Krebs-Ringer-phosphate-HEPES (KRPH) buffer with 2% bovine serum albumin (BSA). Glucose transport was analyzed following the addition of 10 μL of 10 mM 2-DG to insulin-stimulated cells and to noninsulin-stimulated control cells for 20 min. 2-DG is metabolized by cells into 2-DG-6-phosphate (2-DG6P), which results in the oxidation of a substrate. The oxidized substrate was detected at OD412 nm. Data were corrected for protein content, and the protein concentrations of the protein lysates were measured by a Pierce™ BCA Protein Assay kit (Thermo Fisher Scientific).

### Oxidative stress detection

Gastrocnemius SkM and C2C12 myotubes were homogenized in ice-cold lysis buffer. The levels of reduced glutathione (GSH) and oxidized glutathione (GSSG) in the lysates and serum were measured using glutathione assay kits (Meimian, Yancheng, China). The content of H_2_O_2_ in lysates and serum were measured using an enzyme-linked immunosorbent assay kit (Meimian).

### Immunohistochemistry analysis

Freshly harvested gastrocnemius SkM was fixed in 4% paraformaldehyde and embedded in paraffin, and sectioned into 5-μm-thick sections onto poly-l-lysine-coated glass slides. The sections were incubated overnight with primary antibodies against Txnip (Proteintech Group, IL, USA, 18243-1-AP, 1:100), Keap1 (sc-514914, 1:100), and Nrf2 (16396-1-AP, 1:200). Immunostaining was visualized with 3,3-diaminobenzidine substrate.

### Immunofluorescence staining

The sections were incubated with primary antibodies against Irs1 (sc-559, 1:100) overnight at 4 °C. Thereafter, the sections were incubated with Cy3-conjugated goat anti-rabbit secondary antibodies (Wuhan Boster Company, Wuhan, China, 1:100) for 1 h, followed by a 5-min incubation with DAPI (Beyotime Biotechnology, Haimeng, China). Conventional epifluorescence microscopy was used to capture for images (Leica DM2700 P, Germany).

### Transmission electron microscope (TEM)

Gastrocnemius SkM was cut into small pieces, fixed in 2.5% glutaraldehyde for 2 h at 4 °C, and postfixed in 1% osmium tetroxide for 1 h at 4 °C. The tissue was further dehydrated with graded alcohol, embedded in resin, and cut using an ultramicrotome (Leica, German). The ultrathin sections (60-80 nm) were mounted on copper grids, stained with uranyl acetate and lead citrate, and then observed with a transmission electron microscope (HT-7800, Hitachi, Japan). The size of mitochondria was measured using ImageJ software (National Institutes of Health, DC, USA).

### mRNA and miRNA expression

Total RNA, including miRNA, was extracted using TRIzol (Thermo Fisher Scientific), and real-time PCR was carried out by SYBR Green Real-Time PCR Master Mix (Toyobo, Osaka, Japan) on a StepOne Real-Time Quantitative PCR system (Thermo Fisher Scientific). The primers were listed in **Tables S2 and S3**.

### Western blot analysis

Total protein was prepared using RIPA (Thermo Fisher Scientific). The plasma membrane protein was prepared using a membrane protein extraction kit (Thermo Fisher Scientific) and the tissue nuclear protein was prepared using NE-PER nuclear and cytoplasmic extraction reagents (Thermo Fisher Scientific). The protein was separated by electrophoresis on SDS-polyacrylamide gels and immunoblotted with antibodies against pAkt^ser473^ (Cell Signaling Technology, MA, USA, #4060, 1:500), Akt (CST, #9272, 1:2000), Glut4 (sc-53566, 1:500), Insr (CST, #3025, 1:1000), pInsr^Tyr1150/1151^ (CST, #3024, 1:500), Irs1 (sc-515017, 1:500), pIrs1^Tyr608^ (Merck Millipore, 09-432, 1:1000), Txnip (CST, #14715, 1:1000), Keap1 (CST, #8047, 1:1000), Nrf2 (Abcam, ab137550, 1:2000), Dnmt3a (CST, #3598, 1:1000) , Lamin B1 (CST, #13435, 1:1000) and Gapdh (CST, #2118, 1:2000). The antibody-reactive bands were visualized using ECL chemiluminescence detection system (WesternBright™ ECL Western Blotting HRP Substrate, Advansta, CA,USA) and the band intensity was quantified by Image J software (National Institutes of Health).

### Statistical analysis

All data were presented as the mean ± SEM. Comparisons between two groups were performed by two-tailed Student's t test. Multiple comparisons were performed using one-way ANOVA followed by the Bonferroni post hoc test. Statistical analyses were performed using GraphPad Prism 7. The data were considered significant when *P* <0.05 or *P* < 0.01.

## Results

### Exposure to DEHP induced IR

**Figure 1:**
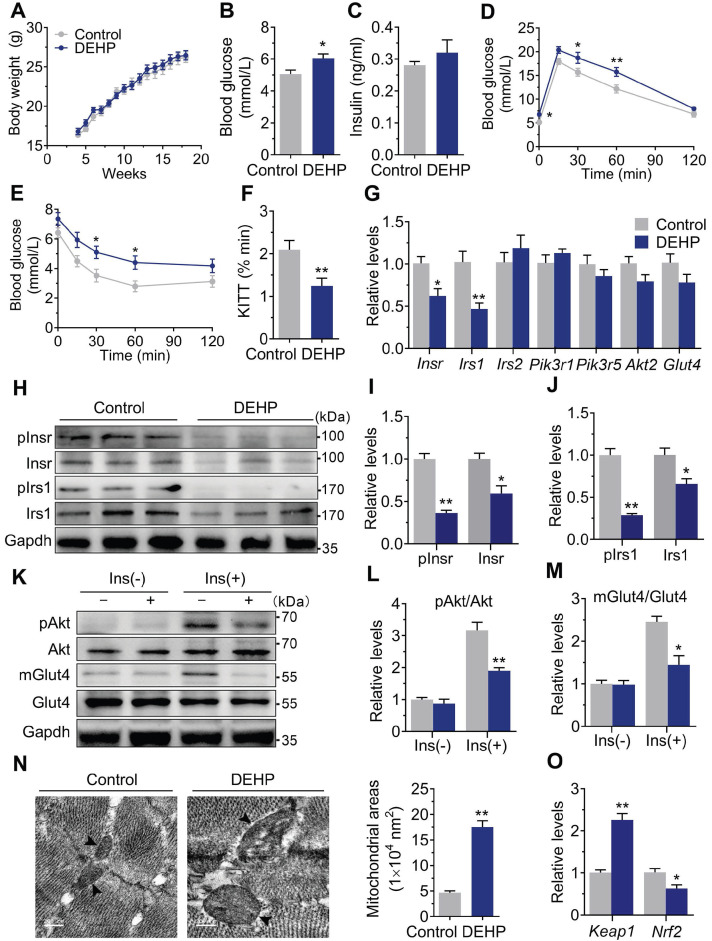
** Exposure to DEHP induced IR. A**. The weekly body weight (n = 10 mice per group). **B**. The fasting blood glucose (n = 10 mice per group). **C**. The fasting serum insulin (n = 10 mice per group). **D**. The dynamics of blood glucose curve during intraperitoneal glucose tolerance test (IPGTT, 2 g/kg bw, n = 5 mice per group). The quantification of total area under the curve (AUC) for IPGTT were shown in Figure S1C. **E**. The insulin sensitivity assessed by insulin tolerance test (ITT, 0.75 U/kg, n = 5 mice per group). **F**. The calculated constant rate for glucose disappearance (KITT) from 0 to 30 min of ITT. **G**. The mRNA expression of genes related to insulin signaling pathway in SkM (n = 4 mice per group). *Gapdh* was used as the loading control. **H-J**. The protein expression and phosphorylation of Insr and Irs1 in SkM (n = 3 mice per group). Gapdh was used as the loading control.** K-M**. The representative western blot images (K) and quantification (L-M) of insulin-stimulated phosphorylation of Akt (pAkt, ser473) and Glut4 translocation in SkM (n = 3 mice per group). mGlut4: Glut4 in plasma membrane, Glut4: Glut4 in total homogenate. All quantitative results were normalized by Gapdh. **N**. The representative TEM images and quantification of mitochondrial areas in SkM. Mitochondria were indicated by black arrowheads. The average mitochondrial area was determined by manually circling 15 mitochondria within the SkM per mice (n = 3 mice per group, Scale bar = 200nm). **O**. The mRNA expression of *Keap1* and *Nrf2* in SkM (n = 4 mice per group). Expression level were normalized to* Gapdh*. All data were presented as the mean ± SEM. *P < 0.05 control mice* vs.* DEHP-exposed mice, **P < 0.01 control mice* vs.* DEHP-exposed mice.

**Figure 2:**
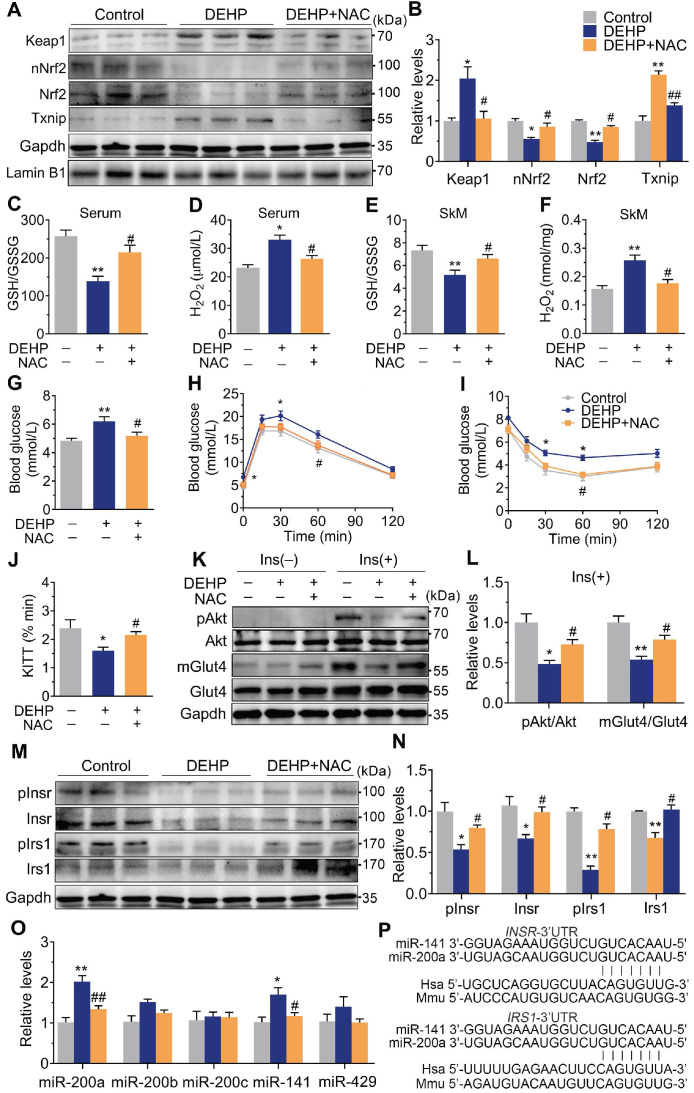
** NAC Prevented DEHP-induced IR. A-B**. The representative western blot images and quantification of oxidative stress-related genes (n = 3 mice per group). The total protein was normalized by Gapdh and the protein expression of Nrf2 in nuclear were normalized by Lamin B1.** C**. The calculated GSH/GSSG ratio in serum (n = 5 mice per group). The serum levels of reduced glutathione (GSH) and oxidized glutathione (GSSG) were shown in Figure S2C-D. **D**. The serum level of H_2_O_2_ (n = 5 mice per group).** E**. The calculated GSH/GSSG ratio in SkM (n = 5 mice per group). The levels of GSH and GSSG in SkM were shown in Figure S2E-F.** F**. The content of H_2_O_2_ normalized to protein content in SkM (n = 5 mice per group). **G**. The fasting blood glucose (n = 10 mice per group).** H**. The IPGTT (n = 5 mice per group). The AUC of the IPGTT were shown in Figure S2G. **I**. ITT (n = 5 mice per group). **J**. The KITT obtained in ITT (0-30min).** K-L**. The representative western blot images (K) and quantification (L) of insulin-stimulated pAkt and the Glut4 translocation in SkM (n = 3 mice per group). The basal levels (without insulin stimulation) of pAkt and mGlut4 was shown in Figure S2H.** M-N**. The expression and phosphorylation of Insr and Irs1 in SkM. Quantitative results were normalized by Gapdh (n = 3 mice per group). **O**. The expression of miR-200 family in SkM of mice (n = 4 mice per group). U6 was used to normalized miRNA expression. **P**. The putative sequence interactions between miR-200a (miR-141) and 3'UTR of *Insr* and *Irs1*, respectively. Mmu, mouse; Hsa, human. All data were presented as the mean ± SEM. *P < 0.05 control mice *vs.* DEHP-exposed mice, **P < 0.01 control mice *vs.* DEHP-exposed mice. #*P* < 0.05 DEHP-exposed mice* vs.* DEHP-exposed mice co-treated with NAC, ##*P* < 0.01 DEHP-exposed mice* vs.* DEHP-exposed mice co-treated with NAC.

As shown in **Figure 1A**, there was no difference in body weight during the 15-wk exposure period. The fasted blood glucose was increased in DEHP-exposed mice (**Figure 1B**), whereas no difference was shown in fasting serum insulin (**Figure 1C**). GTT showed prolonged elevation of blood glucose at 30 and 60 min (**Figure 1D**) and higher glucose AUC (**Figure S1C**) in DEHP-exposed mice. When challenged with ITT, the decrease in blood glucose was less pronounced in DEHP-exposed mice at 30 and 60 min after administration of insulin than in control mice (**Figure 1E**). Moreover, lower glucose decay constant rate (KITT) during the ITT was shown in DEHP-exposed mice (**Figure 1F**), indicating a decreased systemic insulin sensitivity. In addition, the homeostasis model assessment of IR (HOMA-IR) and the quantitative insulin sensitivity check index (QUICKI) were further calculated and the results consistently showed that HOMA-IR was higher, whereas the QUICKI was lower in mice exposed to DEHP compared with control mice (**Figure S1A-B**), indicative of whole-body IR. In SkM, DEHP decreased the expression and phosphorylation of Insr and Irs1 (**Figure 1G-J**). And in response to insulin stimulation, decreased phosphorylation of Akt at Ser473 and defective Glut4 trafficking were also shown in SkM of DEHP-exposed mice (**Figure 1K-M**). Additionally, mitochondria in SkM of DEHP-exposed mice were structurally swollen, enlarged and rounded with disorganized cristae and vacuolar structure (**Figure 1N**). The mRNA and protein expression of Keap1 was increased whereas both total and nuclear protein level of Nrf2 were decreased in SkM of DEHP-exposed mice (**Figure 1O and Figure 2A-B**). The dysregulated Keap1-Nrf2 system was abrogated by NAC administration (**Figure 2A-B**), implying a link between oxidative stress and SkM-IR.

### Oxidative stress acted as an insulin desensitizer in SkM and contributed to DEHP-induced IR

Exposure to DEHP reduced GSH/GSSG ratio and increased H_2_O_2_ in both serum (**Figure 2C,D**) and SkM (**Figure 2E,F**). These changes were accompanied by upregulation of Txnip (**Figure 2A,B**). NAC administration reversed the increased H_2_O_2_ production, reduced GSH/GSSG ratio, impaired Keap1-Nrf2 pathway and increased Txnip in SkM of DEHP-exposed mice (**Figure 2A, B, E and F**). In parallel with protection against oxidative stress, NAC restored hyperglycemia and improved glucose tolerance and insulin sensitivity in DEHP-exposed mice (**Figure 2G-J**). No difference in body weight and serum insulin was observed among control, DEHP-exposed and DEHP and NAC co-treated mice (**Figure S2A-B**). The decreases in Insr, Irs1 and insulin-stimulated pInsr, pIrs1, pAkt and mGlut4 in SkM of DEHP-exposed mice was reversed after cotreatment with the NAC (**Figure 2K-N**).

### miR-200a suppressed insulin signaling in SkM cells by targeting the 3′UTR of *Insr* and *Irs1*

**Figure 3:**
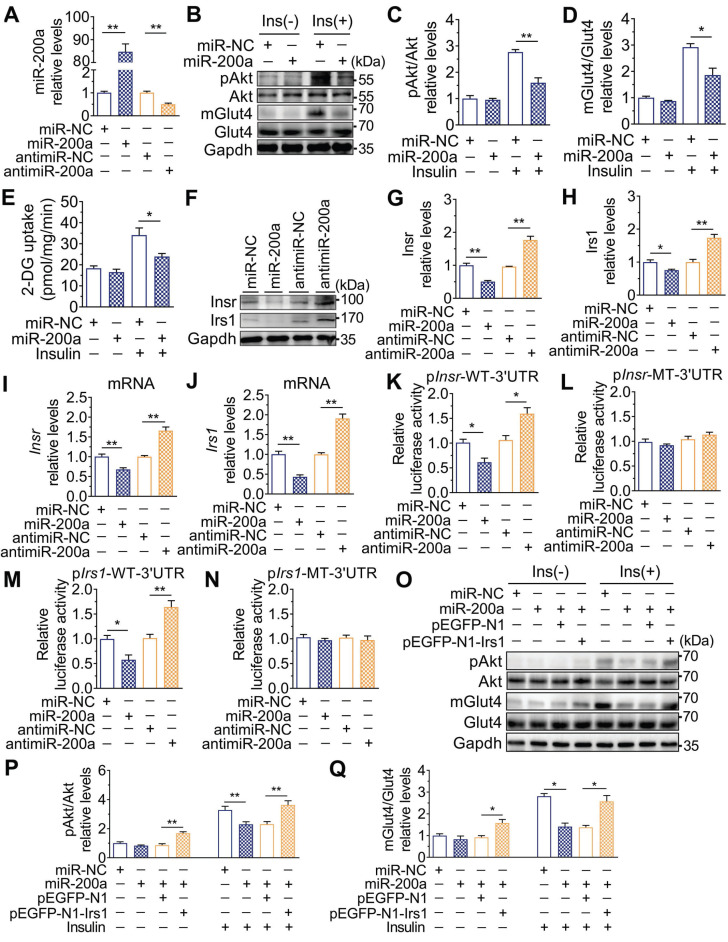
** Upregulation of miR-200a impaired insulin sensitivity by targeting Insr and Irs1 in SkM cells.** C2C12 myotubes were transfected with 50 nM agomiR-200a or 200 nM antagomir-200a for 48 h. **A**. The expression of miR-200a normalized by U6 (n = 4 independent experiments).** B-D**. The insulin-stimulated pAkt and the Glut4 translocation in miR-200a-overexpressing and miR-200a-inhibiting C2C12 myotubes (n = 3 independent experiments). Gapdh was used as the loading control. **E**. The 2-deoxyglucose (2-DG) uptake in miR-200a-overexpressing and miR-200a-inhibiting C2C12 myotubes (n = 3 independent experiments).** F-J**. The protein (F-H) and mRNA (I-J) expression of Insr and Irs1. Gapdh was used as the loading control (n = 3 independent experiments).** K and M**. The relative luciferase activity in C2C12 myoblasts transfected with reporter vector containing the wild* Insr* (H)* or Irs1* (J) 3'UTR together with agomiR-200a, antagomiR-200a or corresponding control (n = 4 independent experiments). The location of the miR-200a binding sites were shown in Figure 2P. **L and N**. The relative luciferase activity in the C2C12 myoblasts transfected with reporter vector containing the mutant Insr (I) or Irs1 (K) 3'UTR together with agomiR-200a, antagomiR-200a or corresponding control (n = 4 independent experiments). Mutated miR-26a binding site was shown in Table S1. **O-Q**. The insulin stimulated Akt phosphorylation and Glut4 translocation in C2C12 myotubes cotransfected with agomiR-200a and transfected with pEGFP-N1-Irs1 plasmid (n = 3 independent experiments). Gapdh was used as the loading control. All data were presented as the mean ± SEM. *P < 0.05, **P < 0.01* vs.* corresponding control as indicated.

The expression of the miR-200 family was examined, and **Figure 2O** showed that miR-200a and miR-141 were increased in SkM of DEHP-exposed mice compared with that of control mice, while NAC inhibited this upregulation. miR-200a and miR-141 have homologous seed regions (**Figure 2P**), and hence, should exhibit similar regulatory modes and have the potential to regulate the expression of same target genes. This study mainly focused on miR-200a. **Figure 3A-E** showed that overexpression of miR-200a in C2C12 myotubes decreased insulin-stimulated pAkt, mGlut4 and glucose uptake. Moreover, overexpression of miR-200a decreased Insr and Irs1, and vice versa (**Figure 3F-J**). Computational algorithms predicted that the 3′UTR of both *Insr* and *Irs1* contained sequences matching the seed sequences for miR-200a (**Figure 2P**). And Figure 3K and M further indicated that overexpression of miR-200a in C2C12 myoblasts decreased luciferase activity of the reporter gene fused with truncated *Insr*- or *Irs1*-3′-UTR sequences containing predicted miR-200a binding site, whereas inhibition of miR-200a had the opposite effects. Both the inhibitory effects of agomiR-200a and the inducible effects of antagomir-200a were abolished when the putative 3′UTR-binding sites were disrupted (**Figure 3L and N**). In addition, **Figure 3O-Q** further showed that miR-200a overexpression failed to inhibit insulin-stimulated pAkt and mGlut4 when Irs1 was overexpressed by pEGFP-N1-Irs1, reinforcing the idea that miR-200a suppressed insulin signaling in SkM cells by targeting Irs1.

### Inhibition of miR-200a improved DEHP-induced IR

**Figure 4:**
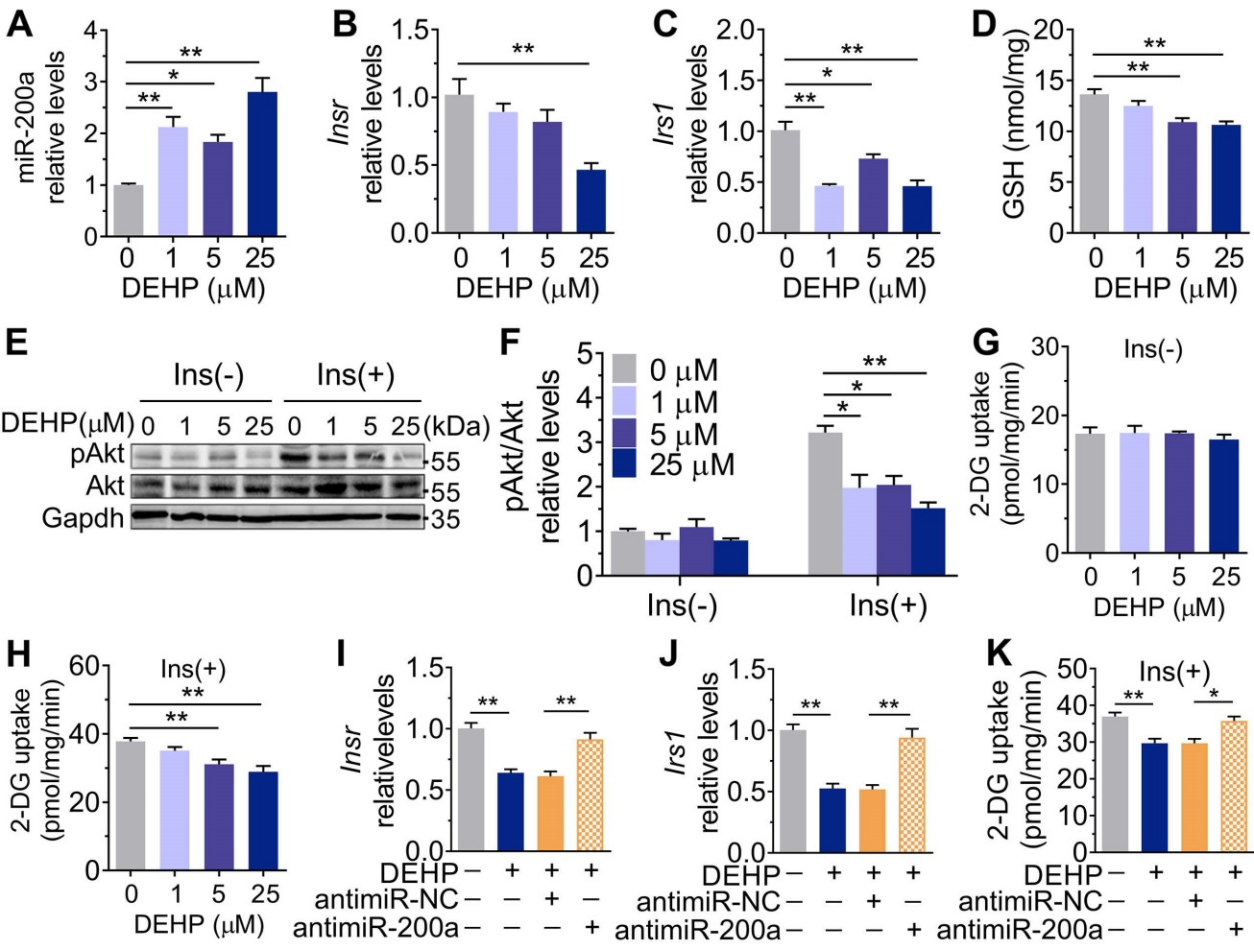
**The role of DEHP exposure in C2C12 myotubes. A-G.** C2C12 myotubes were treated with serial concentrations of DEHP for 48 h (n = 3 independent experiments).** A**. The expression of miR-200a normalized by U6. **B-C**. The mRNA expression of *Insr* (B) and *Irs1*(C) normalized by *Gapdh*.** D**. The content of GSH normalized to protein content in DEHP-exposed C2C12 myotubes. **E-F**. The representative western blot images (**E**) and quantification (**F**) of pAkt in DEHP-exposed C2C12 myotubes. Gapdh was used as the loading control. **G-H**. The basal (G) and insulin-stimulated (H) 2-DG uptake in DEHP-exposed C2C12 myotubes. **I-J**. The mRNA expression of *Insr* (I) and* Irs1* (J) in C2C12 myotubes transfected with 200 nM antagomir-200a and treated with 25 µM DEHP (n = 3 independent experiments). *Gapdh* was used as the loading control.** K**. The insulin-stimulated 2-DG uptake in C2C12 myotubes transfected with 200 nM antagomir-200a and treated with 25 µM DEHP (n = 3 independent experiments). All data were presented as the mean ± SEM. *P < 0.05, **P < 0.01 vs. corresponding control as indicated.

**Figure 5:**
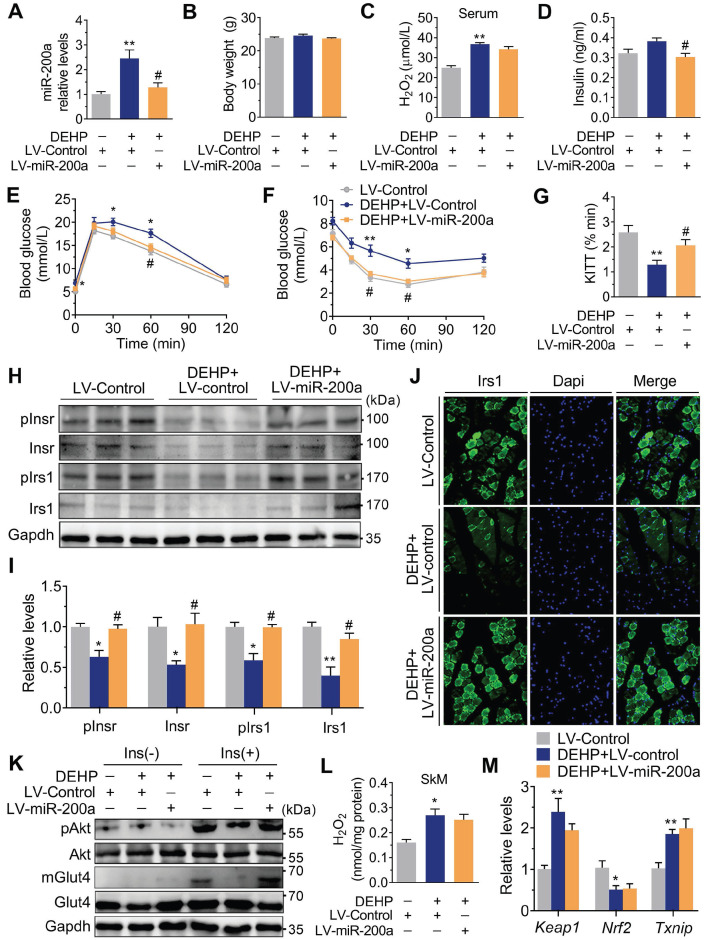
** Inhibition of miR-200a improved DEHP-induced IR.** DEHP-exposed mice were infected with control lentivirus (LV-Control) or anti-miR-200a lentivirus (LV-miR-200a) (n = 6 mice per group). **A**. The expression of miR-200a in SkM. U6 was used to normalized miR-200a expression (n = 3 mice per group). **B**. The body weight (n = 6 mice per group). **C**. The serum level of H_2_O_2_ (n = 6 mice per group). **D**. The fasting serum insulin (n = 6 per group). **E**. The IPGTT (n = 5 mice per group). The calculated AUC of the IPGTT were shown in Figure S4B. **F**. The ITT (n = 5 mice per group). **G**. The KITT obtained in the ITT (0-30min). **H-I**. The expression and phosphorylation of Insr and Irs1 in SkM. Quantitative results (I) were normalized by Gapdh (n = 3 mice per group).** J**. The immunofluorescent detection of Irs1 in SkM (400×, n = 3 mice per group). Nucleus was stained with Dapi (blue) and Irs1 was probed with a primary anti-Irs1 antibody (green). **K**. The insulin stimulated pAkt and mGlut4 in SkM. Quantitative results were normalized by Gapdh and shown in Figure S4C (n = 3 mice per group). **L**. The H_2_O_2_ content normalized to protein content in SkM (n = 3 mice per group). **M**. The mRNA expression of genes related to oxidative stress. *Gapdh* was used as the loading control (n = 3 mice per group). All data were presented as the mean ± SEM. *P < 0.05 DEHP-exposed mice infected with LV-Control* vs.* control mice infected with LV-Control, **P < 0.01 DEHP-exposed mice infected with LV-Control* vs.* control mice infected with LV-Control. #*P* < 0.05 DEHP-exposed mice infected with LV-Control *vs.* DEHP-exposed mice infected with LV-miR-200a.

Consistent with the* in vivo* data, the expression of miR-200a was increased, but *Insr* and *Irs1* were decreased in C2C12 myotubes exposed to serial concentrations of DEHP (**Figure 4A-C**). No obvious evidence of relationship between the dose of DEHP and the miR-200a,* Insr* or *Irs1* expression was observed. Treatment of C2C12 myotubes with DEHP also led to a decrease in GSH (**Figure 4D**), insulin-stimulated pAkt (**Figure 4E,F**) and glucose uptake (**Figure 4G,H**). Both the downregulation of* Insr* and* Irs1* and the inhibition of glucose uptake was prevented in the presence of antagomir-200a (**Figure 4I-K**). *In vivo*, inhibition of miR-200a via LV-miR-200a injection (**Figure 5A**) did not alter body weight (**Figure 5B**), serum H_2_O_2_ (**Figure 5C**) and fasting blood glucose (**Figure S4A**) in DEHP-exposed mice, but fasting serum insulin (**Figure 5D**) was decreased when DEHP-exposed mice received LV-miR-200a. Meanwhile, the GTT and ITT showed that restoration of SkM miR-200a expression improve glucose tolerance and insulin sensitivity in DEHP-exposed mice (**Figure 5E-G**). miR-200a inhibition also abrogated the DEHP-induced downregulation of Insr and Irs1 (**Figure 5H-J**), which was accompanied by pInsr, pIrs1, pAkt and mGlut4 normalization in response to insulin (**Figure 5H, I and K**).

### Impaired Keap1-Nrf2 system and activated Txnip contributed to miR-200a upregulation in DEHP-exposed SkM cells under oxidative stress

Although both NAC and anti-miR-200a lentivirus administration reversed IR in DEHP-exposed mice, the H_2_O_2_ content (**Figure 5L**) and the mRNA expression of *Keap1* and *Nrf2* (**Figure 5M**) were not changed in SkM of DEHP-exposed mice after anti-miR-200a lentivirus injection. In C2C12 myotubes, DEHP increased the expression of *Txnip* (**Figure S6A**), while the loss of *Txnip* decreased miR-200a and enhanced insulin-stimulated glucose uptake in DEHP-exposed myotubes (**Figure S6B-D**). In addition, increased *keap1* and decreased *Nrf2* were observed in DEHP-treated C2C12 myotubes (**Figure S7A,B**). The Nrf2 activator SFN elevated GSH content and reversed DEHP-induced upregulation of miR-200a and *Txnip* in C2C12 myotubes, thereby restoring the defective ability of insulin-stimulated glucose uptake (**Figure S7C-G**).

### Downregulation of miR-17 impaired glucose uptake by inducing oxidative stress via directly targeting keap1

**Figure 6:**
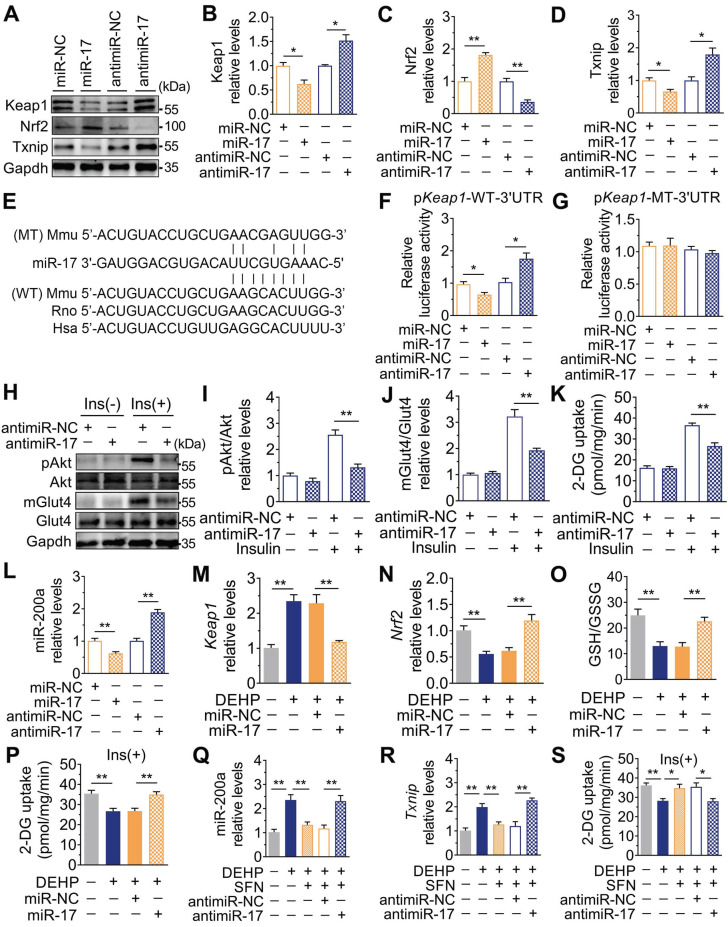
** Downregulation of miR-17 impaired glucose uptake by promoting oxidative stress via targeting keap1. A-D**. The representative western blot images (A) and quantification (B-D) of genes related to oxidative stress. Gapdh was used as the loading control. C2C12 myotubes were transfected with 50 nM agomiR-17 or 200 nM antagomir-17 for 48 h (n = 3 independent experiments). **E**. The miR-17 target regions in 3'UTR of *Keap1*. Mmu, mouse; Rno, rat; Hsa, human. WT: a truncated* Keap1*-3'UTR with wild-type miR-17 binding site; MT: a truncated* Keap1*-3'UTR with mutated miR-17 binding site.** F-G**. The relative luciferase activity in C2C12 myoblasts transfected with reporter vector containing the wild-type (F)* or* mutated (G)* Keap1* 3'UTR together with agomiR-17, antagomiR-17 or corresponding controls (n = 4 independent experiments). **H-J**. The representative western blot images (H) and quantification (I-J) of pAkt and mGlut4 in C2C12 myotubes treated with antagomiR-17 (n = 3 independent experiments). Gapdh was used as the loading control. **K**. The insulin-stimulated 2-DG uptake in C2C12 myotubes treated with antagomiR-17 (n = 3 independent experiments).** L**. The expression of miR-200a in C2C12 myotubes transfected with agomiR-17 and antagomiR-17 (n = 3 independent experiments). U6 was used to normalized miR-200a expression.** M-P**. C2C12 myotubes were transfected with 50 nM agomiR-17 and treated with 25 µM DEHP (n = 3 independent experiments). **M-N**. The mRNA expression of *Keap1* and *Nrf2*.* Gapdh* was used as the loading control. **O**. The calculated GSH/GSSG ratio. The levels of GSH and GSSG were shown in Figure S8C-D.** P**. The insulin-stimulated 2-DG uptake.** Q-S**. C2C12 myotubes were co-treated with 25 µM DEHP, antagomir-17, SFN or corresponding controls (n = 3 independent experiments). **Q**. The expression of miR-200a normalized by U6.** R**. The mRNA expression of *Txnip*. *Gapdh* was used as the loading control. **S**. The insulin-stimulated 2-DG uptake. All data were presented as the mean ± SEM. *P < 0.05, **P < 0.01* vs.* corresponding control as indicated.

**Figure 7:**
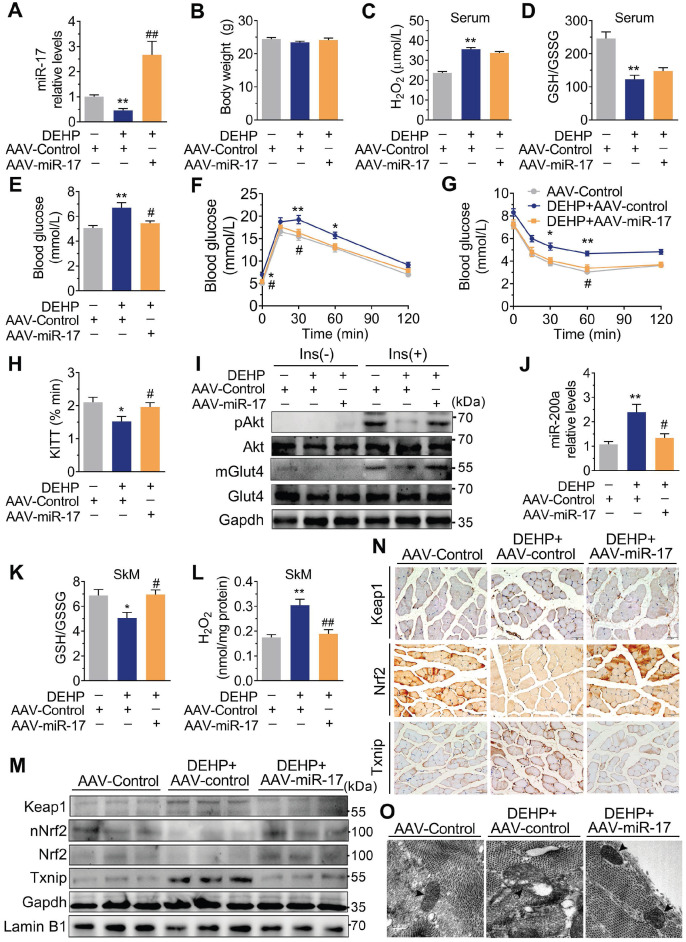
** Overexpression of miR-17 in SkM was resistant to DEHP-induced oxidative stress and IR.** The miR-17 was overexpressed in SkM of DEHP-exposed mice using recombinant adeno-associated virus 9 (AAV9) delivery method (n = 6 mice per group).** A**. The expression of miR-17 in SkM. U6 was used to normalized miR-17 expression (n = 3 mice per group). **B**. The Body weight (n = 6 mice per group). C. The serum level of H_2_O_2_ (n = 5 mice per group). **D**. The GSH/GSSG ratio calculated by the serum levels of GSH (Figure S9B) and GSSG (Figure S9C) (n = 5 mice per group).** E**. The fasting blood glucose level (n = 6 mice per group). **F**. The IPGTT (n = 5 mice per group). The calculated AUC of the IPGTT were shown in Figure S9D. **G**. The ITT (n = 5 mice per group). **H**. The KITT obtained in the ITT (0-30min). **I**. The representative western blot images of pAkt and mGlut4 in SkM (n = 3 mice per group). Gapdh was used as the loading control and the quantification data were shown in Figure S9E-F. **J**. The expression of miR-200a in SkM. U6 was used to normalized miR-200a expression (n = 3 mice per group). **K**. The GSH/GSSG ratio in SkM (n = 3 mice per group). Levels of GSH and GSSG were shown in Figure S9G-H, respectively. **L**. The H_2_O_2_ content in SkM (n = 3 mice per group). **M**. The representative western blot images of genes related to oxidative stress (n = 3 mice per group). The total protein was normalized by Gapdh and the protein expression of Nrf2 in nuclear were normalized by Lamin B1. The quantification data were shown in Figure S9I. **N**. The representative images of immunohistochemical staining of genes related to oxidative stress. (Scale bar = 50 µm). **O**. The representative TEM images of SkM (Scale bar = 200nm, n = 3 mice per group). Mitochondria were indicated by black arrowheads. Calculation of mitochondrial area was shown in the Figure S9J. All data were presented as the mean ± SEM. *P < 0.05 control mice infected with AAV-Control* vs.* DEHP-exposed mice infected with AAV-Control, **P < 0.01 control mice infected with AAV-Control* vs.* DEHP-exposed mice infected with AAV-Control. #*P* < 0.05 DEHP-exposed mice infected with AAV-Control *vs.* DEHP-exposed mice infected with AAV-miR-17, ##*P* < 0.01 DEHP-exposed mice infected with AAV-Control *vs.* DEHP-exposed mice infected with AAV-miR-17.

In addition to changes in miR-141 and miR-200a, exposure to DEHP decreased the expression of miR-17 both* in vitro* and* in vivo* (**Figure S8A and Figure 7A**). Inhibition of miR-17 upregulated Keap1 and Txnip, and downregulated Nrf2 in C2C12 myotubes; whereas overexpression of miR-17 produced the opposite effect (**Figure 6A-D and Figure S8B**). Moreover, the 3′UTR of *Keap1* contained a binding site complementary to miR-17 (**Figure 6E**), and **Figure 6F-G** showed that miR-17 negatively regulated luciferase activity of the wild-type *Keap1* 3′UTR luciferase reporter but not that of the mutant reporter. Consistent with the regulation of oxidative stress, inhibition of miR-17 reduced insulin-stimulated pAkt, mGlut4 and glucose uptake in C2C12 myotubes (**Figure 6H-K**), showing that miR-17 is a positive mediator of insulin signaling. The interaction between miR-17 and miR-200a was also investigated, and **Figure 6L** showed that miR-17 negatively regulated miR-200a in C2C12 myotubes. Overexpression of miR-17 also abrogated the decreased glucose uptake in miR-200a-overexpressed C2C12 myotubes in response to insulin (**Figure S8E**). And inhibition of miR-17-induced miR-200a upregulation was partly abrogated by SFN and siTxnip (**Figure S8F,G**). However, miR-17 expression was independent of miR-200a (**Figure S8H**).

### Overexpression of miR-17 in SkM conferred resistance to DEHP-induced oxidative stress and IR

As shown in **Figure 6M-O**, overexpression of miR-17 in DEHP-exposed C2C12 myotubes revealed parallel downregulation of Keap1, upregulation of Nrf2, and increased GSH/GSSG ratio. Likewise, overexpression of miR-17 increased insulin-stimulated glucose uptake in DEHP-exposed C2C12 myotubes (**Figure 6P**). SFN failed to reduce miR-200a and oxidative stress and no longer restored glucose uptake in DEHP-exposed C2C12 myotubes when miR-17 was knock down (**Figure 6Q-S**). In DEHP-exposed mice, the SkM-specific overexpression of miR-17 using AAV9 (**Figure 7A**) did not affect body weight and serum insulin (**Figure 7B and Figure S9A**). No significant difference was observed in serum H_2_O_2_ level and GSH/GSSG ratio between DEHP-exposed mice receiving AAV-control and those receiving AAV-miR-17 (**Figure 7C-D**), suggesting that AAV9-mediated SkM-specific overexpression of miR-17 could not alter DEHP-induced system oxidative stress. However, SkM-specific overexpression of miR-17 resulted in a significant improvement in higher blood glucose (**Figure 7E**), glucose intolerance (**Figure 7F**), and insulin insensitivity (**Figure 7G,H**), and moreover, rescued the SkM from the suppression of insulin-stimulated pAkt and mGlut4 (**Figure 7I**). AAV-miR-17 also downregulated miR-200a in SkM of DEHP-exposed mice (**Figure 7J**). For redox homeostasis, exogenous expression of miR-17 reduced GSH/GSSG ratio (**Figure 7K**) and H_2_O_2_ content (**Figure 7L**), activated Keap1-Nrf2 pathway and decreased Txnip in SkM of DEHP-exposed mice (**Figure 7M,N**). Additionally, mitochondrial swelling and vacuolization in SkM were not present when DEHP-exposed mice were injected with AAV-miR-17 (**Figure 7O**).

### Dnmt3a and lncRNA Malat1 cooperatively suppressed miR-17 in SkM

**Figure 8:**
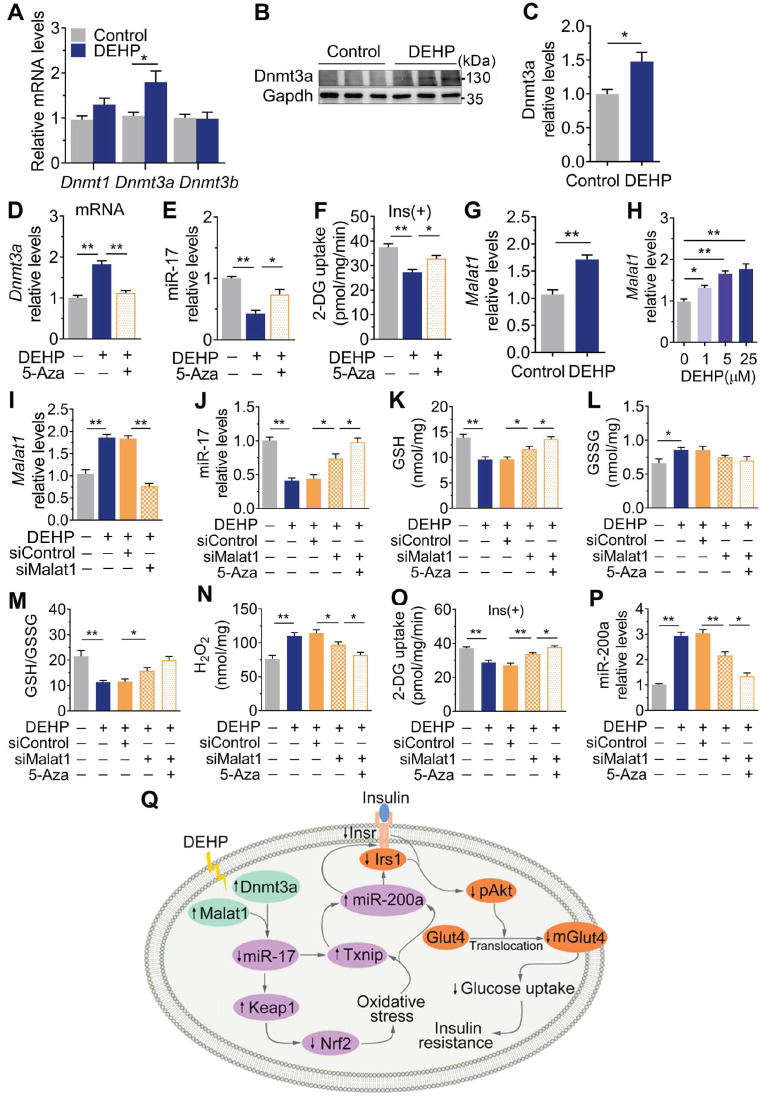
** Dnmt3a and lncRNA Malat1 cooperatively suppressed miR-17 in SkM. A-C**. The mRNA (A) and protein (B-C) levels of DNA methyltransferase in SkM of DEHP-exposed mice (n = 4 mice per group qRT-PCR analysis and n = 3 mice per group for western blot). Gapdh was used as the loading control. **D**. The mRNA expression of* Dnmt3a* in DEHP-exposed C2C12 myotubes co-treated with 5-Aza (n = 3 independent experiments). *Gapdh* was used as the loading control.** E**. The expression of miR-17 in DEHP-exposed C2C12 myotubes co-treated with 5-Aza (n = 3 independent experiments). U6 was used to normalized miR-17 expression.** F**. The insulin-stimulated 2-DG uptake in DEHP-exposed C2C12 myotubes co-treated with 5-Aza (n = 3 independent experiments).** G-H**. The expression of lncRNA Malat1 in SkM of DEHP-exposed mice (G, n = 4 mice per group) and DEHP-treated C2C12 myotubes (H, n = 3 independent experiments).* Gapdh* was used as the loading control. **I**. The expression of lncRNA Malat1 in C2C12 myotubes transfected with lncRNA Malat1 siRNAs and treated with 25 µM DEHP (n = 3 independent experiments). *Gapdh* was used as the loading control.** J-O**. C2C12 myotubes were co-treated with 25 µM DEHP, 5-Aza, *Txnip* siRNAs or corresponding control (n = 3 independent experiments).** J**. The expression of miR-17 normalized by U6.** K**. The GSH content normalized to protein content in C2C12 myotubes.** L**. The GSSG content normalized to protein content in C2C12 myotubes. **M**. The calculated GSH/GSSG ratio. **N**. The H_2_O_2_ content. **O**. The insulin-stimulated 2-DG uptake. **P**. The expression of miR-200a normalized by U6. **Q**. The proposed signaling pathway involved in DEHP-induced IR. All data were presented as the mean ± SEM. *P < 0.05, **P < 0.01* vs.* corresponding control as indicated.

**Figure 8A-C** showed that exposure to DEHP increased the level of the DNA methyltransferases Dnmt3a in SkM of mice. 5-Azacytidine (5-Aza), a synthetic Dnmt inhibitor, decreased Dnmt3a and partly increased miR-17 and insulin-stimulated glucose uptake in DEHP-exposed C2C12 myotubes (**Figure 8D-F**). Notably, the decrease in miR-17 was not completely corrected by 5-Aza in DEHP-exposed C2C12 myotubes, so an additional mechanism might be involved in regulating miR-17 in SkM under DEHP exposure. LncRNAs function as ceRNAs to sponge miRNAs, and **Figure 8G-H** showed that DEHP increased the expression of lncRNA Malat1 in SkM cells both *in vivo* and *in vitro*. 5-Aza treatment together with lncRNA Malat1 siRNA fully antagonized the decreases in miR-17, GSH/GSSG ratio and insulin-stimulated glucose uptake in DEHP-exposed C2C12 myotubes, and the increases in H_2_O_2_ (**Figure 8I-O**). Unlike miR-17, a decrease of miR-200a expression was showed in DEHP-exposed C2C12 myotube when cotreated with 5-Aza and lncRNA Malat1siRNA and either lncRNA Malat1siRNA (**Figure 8P**).

## Discussion

Exposure to DEHP is a potent contributor to the development of T2D. Our current study indicated that DEHP decreased Insr and Irs1 in SkM of mice. Downregulation of Insr and Irs1 by DEHP led to deactivation of Akt in response to insulin, which subsequently suppressed glucose uptake by decreasing mGlut4 in SkM, ultimately promoting the development of whole-body IR. Although this study set up an DEHP-exposed animal model to study SkM-IR and whole-body glucose homeostasis, we further found that 18-week high-fat diet (40.86% fat, 21.24% protein, and 37.9% carbohydrates, with energy of 4.398 kJ/g) feeding induced similar changes in miR-17, Keap1, Nrf2 and miR-200a expression patterns in SkM in male C57BL/6 mice (**Figure S10**), compared with the mice model of DEHP-triggered IR. Moreover, regulation and biological function of miR-17 and miR-200a in regulating insulin-stimulated pAkt, mGlut4 and glucose uptake via Keap1-Nrf2 and Irs1/Insr were also demonstrated in C2C12 myotubes. Therefore, we suggested that it would be possible to extend this work to other models of IR. Of course, it should be also noted that more supplementary experiments are still needed to test whether the role of miR-17/Keap1-Nrf2/miR-200a cascade could appropriately work in other animal models of SkM-IR.

In this study, we identified for the first time that both Insr and Irs1 were direct targets of miR-200a that control insulin signaling in C2C12 myotubes and suggested a function of miR-200a in promoting SkM-IR. The miR-200 family has been linked to the disorders of glucose homeostasis as an adverse factor. The miR-200 family was upregulated in pancreatic islets of 12-week-old db/db mice 22. Moreover, β cell-specific overexpression of miR-200c triggered β cells apoptosis and T2D, and conversely, ablation of miR-200c ameliorated T2D in mice 22. miR-200a, miR-200b and miR-429 were also reported to be upregulated in the hypothalamus of ob/ob mice, and the hypothalamic silencing of miR-200a increased Irs2 and restored liver insulin responsiveness 23. However, there were also paradoxical changes in miR-200a coexisting in liver tissues during diabetes 24, 25, which unveiled the possibility that miR-200a was expressed in a tissue-selective manner during metabolic disturbance. In SkM, this study supported the facilitating role of miR-200a in IR by showing that DEHP-decreased glucose uptake resulted from the miR-200a-impaired insulin signaling. Injection of anti-miR-200a lentivirus abrogated the downregulation of Insr and Irs1 and restored insulin-dependent pAkt and Glut4 translocation in SkM of DEHP-exposed mice, consequently ameliorating whole-body IR. However, we noted that the blood glucose remains unchanged and serum insulin was decreased in DEHP-exposed mice after LV-miR-200a injection. We speculated that this is most probably because administration of lentiviruses resulted in transgene expression in several tissues other than SkM. For instance, hepatic miR-200a downregulation would inhibit the activation of the Akt/Gsk pathway and decreased the glycogenesis in the hepatocytes 25. Under this circumstance, some unpredicted results may be obtained. As a result, accurate determination of miRNA expression patterns in different tissue and cell types is essential to achieve miRNA-based therapy 32. Improving defects in glucose uptake by SkM-specific correction of miR-200a expression would be a good way to prevent the development of T2D at its earliest stages.

In this study, no evidence of relationship between the dose of DEHP and the expression of miR-200a, Insr and Irs1 in C2C12 myotubes was observed. Presently, we could only offer some speculation. Firstly, the hazard and risk assessment of chemicals with endocrine activity is hotly debated partly due to non-monotonous dose-response, and there were evidence suggesting that EDCs have non-linear dose-response effects 33, 34. The dose-response relationships between urinary phthalate metabolites and thyroid hormone parameters were reported to be non-monotonic among the waste plastic recycling workers 35. And there were also studies indicating non-linear associations of phthalate metabolites (∑DEHP) with IR indices 36. Metabolic reactions could be partly responsible for the non-linear dose-response relationships of DEHP 37. DEHP absorbed in the body is metabolized into MEHP and *in vitro* cultured cells also have the capacity to convert of DEHP to its metabolites 38. Enzymes involved in DEHP metabolism might display non-linear concentration-velocity relationships with respect to substrates or co-factors, resulting in different efficiency of metabolic activation and detoxification of DEHP. In addition, higher-dose exposure would also affect other regulatory pathways, and the response to these pathway and factors might also counteract or interfere with the stimulation produced by DEHP at miR-200a, Insr and Irs1 expression. Finally, a limitation of this *in vitro* experimental design was the relatively narrow-range exposure dose. It seemed not to be suitable for dose-response analyses. Experiments varied the exposure doses over a wider range are need for further dose-response study of DEHP.

In this study, a reduction/oxidation imbalance was shown in SkM of DEHP-exposed mice. The cross-talk between the miR-200 family and redox homeostasis modulation has been studied, and H_2_O_2_ was reported to upregulate miR-200 family in HUVECs, C2C12 myoblasts 19, 39 and liver cells 21. Upregulation of the miR-200 family, especially miR-200c, in turn, further enhanced oxidative stress. Oxidative stress plays a central role in the pathogenesis of IR and diabetes, which promotes the use of antioxidants as a complementary therapeutic approach. In this study, the onset of hyperglycemia and IR was indeed alleviated in DEHP-exposed mice after NAC treatment, which was parallel to the decreases in miR-200a. However, it was interesting, that anti-miR-200a lentivirus did not alleviate DEHP-increased H_2_O_2_ in the SkM. miR-200a might be a target or downstream mediator of redox imbalance in SkM during IR.

Keap1-Nrf2 signaling is a key antioxidant system that can detoxify exogenous toxicants to maintain cellular homoeostasis 17, 40, 41. Decreased Nrf2 rendered cells susceptible to oxidative stress. Previously, we found that DEHP deactivated the Nrf2-mediated antioxidant response in INS-1 cells, resulting in insulin-secretion deficiency 8. Similarly, Nrf2 was downregulated by DEHP in SkM, associated with increased Keap1. The relationship between miR-200 family and Keap1-Nrf2 signaling is a matter of great concern, and there were some studies showing that miR-200a regulated Nrf2 activation by targeting Keap1 42-44. Actually, in our previous study, we also reported that miR-200a and miR-141 acted to target Keap1 in renal mesangial cells, resulting in profound dysregulations in Keap1-Nrf2 signaling during the development of diabetic nephropathy. However, this current study unexpectedly found that miR-200a did not regulate Keap1-Nrf2 signaling in SkM and C2C12 myotubes (**Figure S5**). Instead, the expression of miR-200a, along with the impaired glucose disposal and insulin signaling, was reversed by SFN treatment in DEHP-exposed C2C12 myotubes. This finding was in agreement with studies reporting that oxidative stress has an effect to upregulate miR-200 family in several cell types 19, 21, 39, 45. We believed that miR-200a acted as an oxidative stress-responsive factor, and the Keap1-Nrf2 antioxidant system played a critical role in maintaining insulin sensitivity in SkM.

Nrf2 is also a key gatekeeper of Txnip transcription, which maintains the low-level basal expression of Txnip 46. Nrf2 alleviated the oxidative damage of cardiomyocytes in diabetic mice by repressing Txnip 46. The absence of Txnip led to excess glucose uptake in muscle, causing hypoglycemia 47. Downregulation of TXNIP in muscle via caloric restriction 48 or exercise training 49 improved insulin signaling and peripheral glucose metabolism in humans. In accordance, DEHP-induced oxidative stress that contributed to increased Txnip in SkM, which was mechanistically associated with diminished activity of insulin-dependent Akt and glucose transport. Txnip potentially played a role in the regulation of several miRNAs including miR-204 50, miR-124a 51 and miR-200 52, that mediated the Txnip-induced inhibition of insulin production and sensitivity under diabetic conditions. With regard to miR-200a, Txnip elevated its expression in pancreatic β cells and thereby promoted β cells apoptosis 52 and this study further identified the direct transcriptional regulatory action of Txnip on miR-200a in SkM.

Apart from miR-200a, DEHP decreased miR-17 in SkM. miR-17 is one of key players in the pathogenesis of T2D 53-57. Serum miR-17 was consistently reported to be downregulated in patients with T2D 58-61. In diabetic mice, expression of miR-17 was also lower in macrophages than normal mice, and upregulation of miR-17 indirectly increased insulin-stimulated glucose uptake by preventing inflammatory cytokine secretion, therefore significantly improved inflammation-induced IR 56. Pancreatic β-cell dysfunction and IR are two major causes for T2D. Conditional deletion of miR-17 in mouse pancreatic β cells significantly reduced glucose tolerance and glucose-stimulated insulin secretion 55, finally promoting the development of experimental diabetes. Insulin-resistant subjects also exhibit decreased insulin sensitivity in major insulin target tissues including liver, adipose tissue and skeletal muscle, and there were studies demonstrating that miR-17-92 cluster was involved in insulin signal transduction in these organs. In obese people, lower expression of miR-17 was detected in omentum fat, which interacted with genes including GLUT4 and so on to regulate insulin sensitivity in adipose tissue and increased fasting blood glucose and glycosylated hemoglobin 53. In a rat model of streptozotocin and high-fat diet-induced T2D, miR-17 family, especially miR-17, was also downregulated in the hepatocytes. Moreover, miR-17 overexpression enhanced insulin sensitivity in HepG2 and LO2 cells, characterized by altered phosphorylation on Insr signaling pathway proteins 62. However, there was a study reporting that miR-17 impaired glucose metabolism by repressing Glut4 in SkM of a high-fat diet STZ-induced rat model and palmitic acid-exposed L6 rat skeletal muscle cell line 63. Our current study supported the antidiabetic role of miR-17, because DEHP-induced glucose intolerance, Akt inactivation, and mGlut4 attenuation were abolished when miR-17 was overexpressed in SkM by AAV-miR-17. Although the expression of Glut4 in antagomiR-17-treated total cell lysates was unaltered following insulin treatment, decreased plasma membrane concentration of Glut4 and the parallel impairment of glucose uptake were shown in C2C12 myotubes under these conditions. Whether the influence of miR-17 on the expression of target genes involved in insulin signal and glucose uptake might differ according to the species and cell types, is at present unclear and remains to be demonstrated in future studies. But importantly, our study further revealed a novel function of miR-17 in physically interacting with Keap1 and Txnip in SkM. Overexpression of miR-17 in C2C12 myotubes inhibited keap1 and Txnip and downregulated miR-200a, which corresponded with the improved pAkt and glucose uptake. Highly conserved seed sequences for miR-17 in the Txnip 3′UTR were previously reported, and Txnip was predictably modulated by miR-17 in INS-1 cells 57. This study discovered that the 3′UTR of Keap1 contained a conserved binding site that matched miR-17 in humans, mice and rats and first identified the direct regulation of Keap1 by miR-17 in C2C12 myotubes. Above all, it was believed that the alteration of Glut4 in either miR-17-knockdown or DEHP-exposed SkM cells was most likely a consequence rather than a cause of SkM-IR. Inhibition of miR-17 upregulated miR-200a to impair Glut4 translocation and muscle-specific glucose uptake in response to insulin signaling by inducing oxidative stress in SkM through direct targeting Keap1 and Txnip, ultimately controlling over whole-body glucose homeostasis. miR-17 acted as an activator, whereas miR-200a acted as an inhibitor that regulated insulin action and glucose homeostasis.

Interestingly, this study found that SFN failed to reduce miR-200a and oxidative stress, and to restore glucose uptake in DEHP-exposed C2C12 myotubes when miR-17 was knockdown, suggesting that antioxidant effects of miR-17 was stronger than those of the small molecule Nrf2 inducer SFN. SFN is an electrophilic entity with very small molecular weight and poor aqueous, and this activator has some additional disadvantages hindering its development as a drug, such as the relatively low potency in functional assays measuring the induction of antioxidant proteins and the activation of ROS-detoxifying enzymes 64. Precision therapeutics to target dysregulated miRNA can be an appropriate target for disease treatment. Some miRNA-based therapies have been entering into the earlier-phase clinical trials 32, 65. As just one example, antimiR against miR-122, miravirsen (Santaris Pharma), has been currently in a Phase II clinical trial (Clinical Trial Numberi: NCT01200420) to treat hepatitis C virus (HCV) patients 66. Therefore, targeting the regulation of the Nrf2 signaling pathway by miR-17 would potentially lead to the development of innovative therapeutic strategies for oxidative stress-induced complications, particularly SkM-IR. On the other hand, intramuscular injection of AAV9-delivered miR-17 resulted in the protection of SkM against oxidative stress and IR, but DEHP-induced changes in serum oxidative stress parameters (H_2_O_2_, GSH and GSSG) were not ameliorated. Indeed, due to the functional complexity, the same miRNA may differ in expression and its targets among different tissues and cell types from the same disease 32. Designing an optimal miR-17 delivery strategy that maintains sufficient molecular stability and enables SkM-specific targeting is particularly important to enable miR-17-based therapeutics in IR and T2D to become a reality.

In this study, exposure to DEHP increased Dnmt3a in SkM of mice. Dnmt3a is a methyltransferase that is responsible for the *de novo* methylation of promoter CpGs with different targets. The expression of miRNA can be regulated by Dnmt3a-dependent promoter methylation 67, 68. In NIH 3T3 fibroblasts, Dnmt3a induced methylation of the miR-17 promoter to decrease its expression 68. Similarly, cotreatment of DEHP-exposed C2C12 myotubes with 5-Aza-induced downregulation of Dnmt3a partly restored miR-17 expression and the subsequent decreases in glucose uptake. Thus, it was reasonable to postulate that the DEHP-induced downregulation of miR-17 might be partially attributed to the Dnmt3a-triggered promoter methylation of miR-17. In addition to epigenetic regulation by methylation, lncRNA Malat1 was shown to function as a competitive endogenous RNA to sponge miR-17. Higher level of lncRNA MALAT1 and lower level of miR-17 were detected simultaneously in patients with diabetes who smoked 69. Knockdown of lncRNA MALAT1 induced an increase in miR-17, which then suppressed TXNIP and promoted the production of insulin in pancreatic β cells 69. In this study, complete recovery of miR-17 and normalized GSH and insulin-stimulated glucose uptake were exhibited in DEHP-exposed C2C12 myotubes after combined treatment with lncRNA Malat1 siRNAs and 5-Aza. Dnmt3a and lncRNA Malat1 cooperatively suppressed miR-17, thereby regulating downstream factors of the Txnip and keap1-Nrf2 pathways related to the oxidative stress and IR. More *in vivo* investigations are required for further exploration. In fact, apart from miR-17, the promoter methylation of miRNA and the sponge role of lncRNA for several miRNAs have been reported. For miR-200a, it was reported that miR-200a-5p 70 and miR-200a 71 could be methylated by Dnmt3a in the process of breast cancer proliferation. Besides, there were studies also reporting that lncRNA Malat1 participated in proliferation, migration, and invasion in human hepatoma Hep3B 72 and non-small cell lung cancer cells 73 via sponging miR-200a. But notably, IR and cancer are totally different pathological and physiological conditions. In this study, miR-200a was decreased after that lncRNA Malat1 and Dnmt3a was inhibited in DEHP-exposed C2C12 myotube. As a result, we suggested that either Dnmt3a or lncRNA Malat1 indirectly regulated miR-200a in insulin-resistant SkM, which should be independent of methylation-associated silence and miRNA sponges, respectively.

In summary, this study identified a novel pathway by which DEHP decreased miR-17 to induce oxidative stress through Keap1-Nrf2 and Txnip. The Dnmt3a-dependent methylation of promoter and lncRNA Malat1-related sponge functions jointly regulated miR-17. In SkM, the induction of oxidative stress upregulated miR-200a which served to inhibit Insr and Irs1, thus decreasing the insulin-mediated activation of Akt and glucose uptake and ultimately resulting in IR (**Figure 8Q**). The signaling node of miR-17/Keap1-Nrf2/miR-200a is a good treatment and prevention option for IR and T2D.

## Supplementary Material

Supplementary figures and tables.

## Abbreviations

2-DG: 2-deoxyglucose
3′UTR: 3′untranslated regions
AAV9: adeno-associated virus 9
DEHP: di(2-ethylhexyl) phthalate
DMEM: dulbecco's modified Eagle medium
EDCs: environmental endocrine-disruptingchemicals
FBS: fetal bovine serum
GSH: glutathione
IR: insulin resistance
Insr: insulin receptor
Irs1: insulin receptor substrate 1
Keap1: kelch like ECH associated protein 1
NAC: n-acetylcysteine
Nrf2: nuclear factor (erythroid-derived 2)-like 2
miRNA: microRNA
GSSG: oxidized glutathione
Akt: serine/threonine kinase
SFN: sulforaphane
SkM: skeletal muscle
Glut4: solute carrier family 2 member 4
TEM: transmission electron microscope
T2D: Type 2 diabetes
