# Supplementary material for: Epigenetic repression of miR-17 contributed to di(2-ethylhexyl) phthalate-triggered insulin resistance by targeting Keap1-Nrf2/miR-200a axis in skeletal muscle
Source: Theranostics. 2020 Jul 23;10(20):9230–48. doi: 10.7150/thno.45253 (PMC7415800; doi:10.7150/thno.45253)
Supplement: Supplementary file 1 — Supplementary figures and tables. [file thnov10p9230s1.pdf]

Epigenetic repression of miR-17 contributed to di(2-ethylhexyl) phthalate-triggered insulin resistance by targeting Keap1-Nrf2/miR-200a axis in skeletal muscle

Jie Wei[1, †,*], Qiongyu Hao[3,†], Chengkun Chen[1], Juan Li[1], Xikui Han[1], Zhao Lei[2], Tao Wang[4], Yinan Wang[1], Xiang You[1], Xiaoxuan Chen[2], Huasheng Li[1], Yuxin Ding[1], Weihao Huang[1], Yangyang Hu[1], Shuirong Lin[1], Heqing Shen[2,*], Yi Lin[2,*]

Supplementary Material

Figures

Figure S1

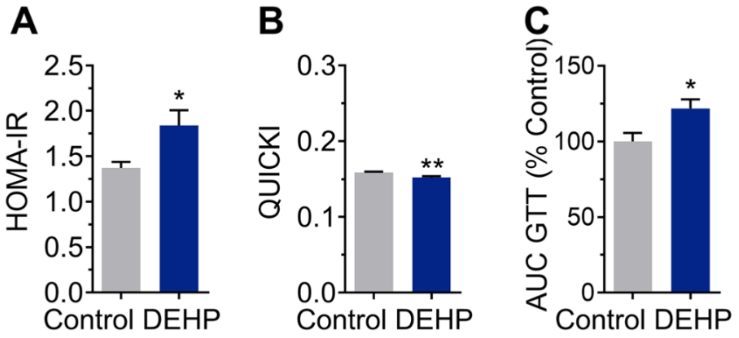

Figure S1 Homeostasis Model Assessment of exposure to DEHP-induced IR. Homeostasis Model Assessment of IR (HOMA-IR) and quantitative insulin sensitivity check index (QUICKI) were calculated from fasting blood glucose and serum insulin values (n = 10 mice per group). **A**. HOMA-IR calculated as fasting glucose (mM) × fasting insulin (μU/mL) /22.5. **B**. QUICKI calculated as 1 / (ln (fasting insulin, (μU/mL) + ln (fasting glucose, mg/dL)). **C**. The total area under the curve (AUC) for IPGTT (Figure 1D) calculated using the trapezoidal method. All data were presented as the mean ± SEM. *P < 0.05 control mice vs. DEHP-exposed mice, **P < 0.01 control mice vs. DEHP-exposed mice.

Figure S2

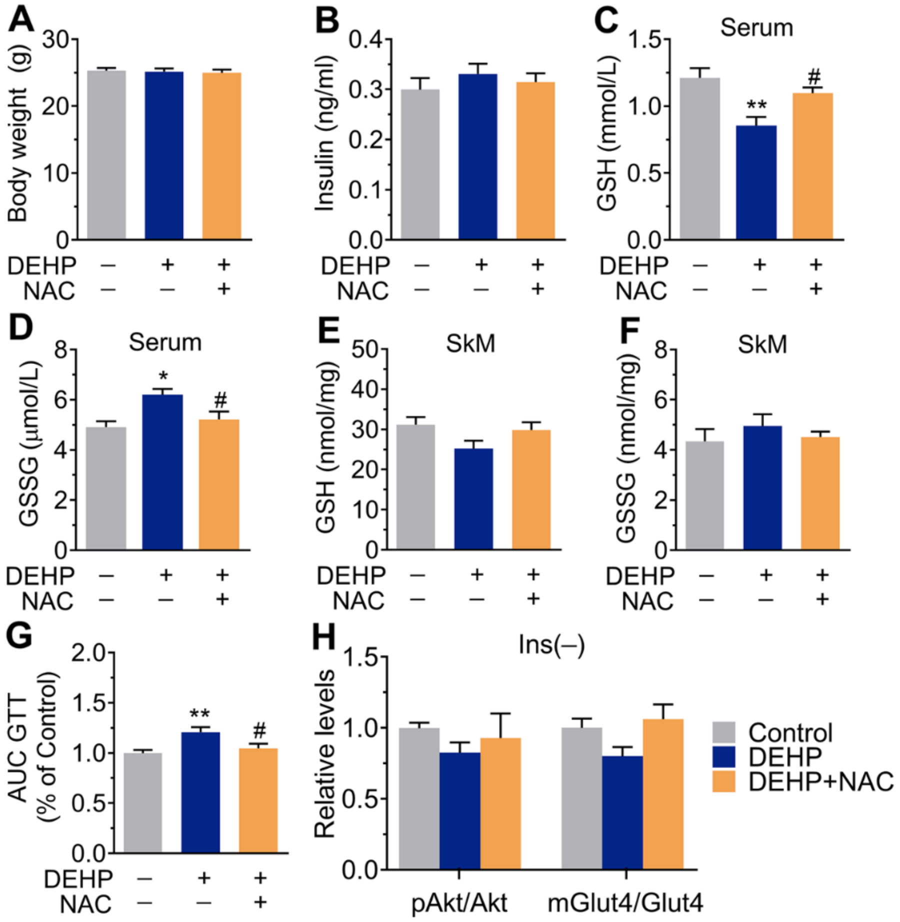

Figure S2 Antioxidant treatment in the DEHP-exposed mice. mice were subjected to 2 mg/kg/day of DEHP dissolved in corn oil by oral gavage for 15 week, and 2 mM of NAC was administered to DEHP-exposed mice in drinking water throughout the experimental period. **A**. The body weight (n = 10 mice per group). **B**. The fasting serum insulin (n = 10 mice per group). **C-D**. The serum levels of reduced glutathione (GSH) and oxidized glutathione (GSSG) (n = 5 mice per group). The calculated GSH/GSSG was shown in Figure 2C. **E-F**. The GSH and GSSG normalized to protein content in SkM (n = 5 mice per group). The calculated GSH/GSSG was shown in Figure 2E. **G**. The AUC of the IPGTT in Figure 2H (n = 5 mice per group). **H**. The quantification for the basal levels (without insulin stimulation) of pAkt and the Glut4 translocation in SkM. Quantitative results were normalized by Gapdh. The representative western blot images were shown in Figure 2K (n = 3 mice per group). All data were presented as the mean ± SEM. *$P < 0.05$ control mice *vs.* DEHP-exposed mice, **$P < 0.01$ control mice *vs.* DEHP-exposed mice. #$P < 0.05$ DEHP-exposed mice *vs.* DEHP-exposed mice co-treated with NAC, ##$P < 0.01$ DEHP-exposed mice *vs.* DEHP-exposed mice co-treated with NAC.

Figure S3

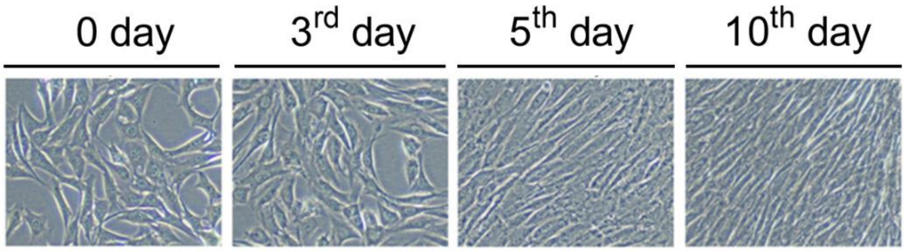

Figure S3 The differentiation of C2C12 myoblasts (0-10 day). When the C2C12 myoblasts reached 80% confluence, the cells were switched to differentiation medium consisting of DMEM supplemented with 2% horse serum. Myotubes were used for experiments after 6 days of differentiation.

Figure S4

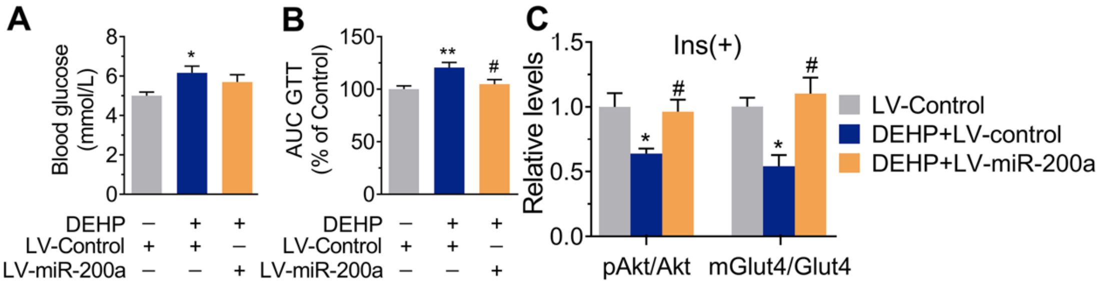

Figure S4 Inhibition of miR-200a improved DEHP-induced IR. DEHP-exposed mice were infected with control lentivirus (LV-Control) or anti-miR-200a lentivirus (LV-miR-200a). **A**. The fasting blood glucose (n = 6 mice per group). **B**. The AUC of the IPGTT shown in Figure 5E (n = 5 mice per group). **C**. The quantification for insulin-stimulated pAkt and mGlut4 in SkM (n = 3 mice per group). Quantitative results were normalized by Gapdh. The representative western blot images were shown in Figure 5K. All data were presented as the mean ± SEM. **P* < 0.05 DEHP-exposed mice infected with LV-Control *vs.* control mice infected with LV-Control, ***P* < 0.01 DEHP-exposed mice infected with LV-Control *vs.* control mice infected with LV-Control. #*P* < 0.05 DEHP-exposed mice infected with LV-Control *vs.* DEHP-exposed mice infected with LV-miR-200a.

Figure S5

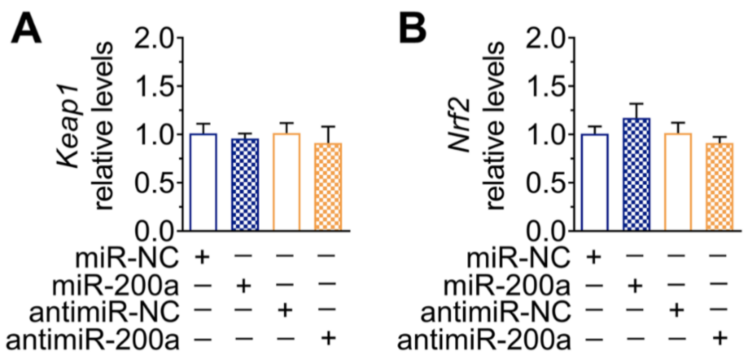

Figure S5 The role of miR-200a on Keap1-Nrf2 signaling in C2C12 myotubes. The C2C12 myotubes were transfected with 50 nM agomiR-200a or 200 nM antagomir-200a for 48 h (n = 3 independent experiments). **A**. The mRNA expression of Keap1 normalized by *Gapdh*. **B**. The mRNA expression of *Nrf2* normalized by *Gapdh*. All data were presented as the mean ± SEM.

Figure S6

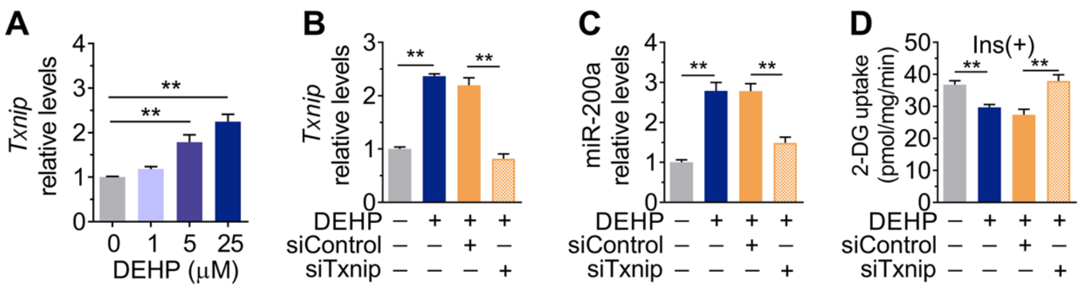

Figure S6 The effects of Txnip inhibition on DEHP-treated C2C12 myotubes. A. The mRNA expression of *Txnip* in DEHP-treated C2C12 myotubes (n = 3 independent experiments). *Gapdh* was used as the loading control. **B-D**. C2C12 myotubes were transfected with si*Txnip* and co-treated with 25 μM DEHP or corresponding controls (n = 3 independent experiments). **B**. The mRNA expression of *Txnip* normalized by Gapdh. **C**. The expression of miR-200a normalized by U6. **D**. The insulin-stimulated 2-DG uptake. All data were presented as the mean ± SEM. **P < 0.01 vs. corresponding control as indicated.

Figure S7

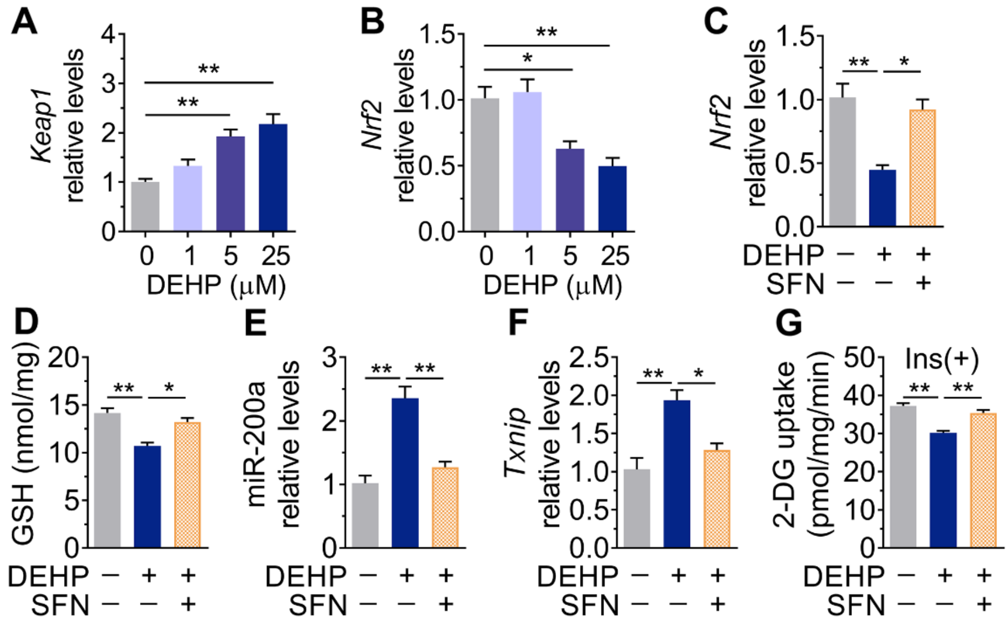

Figure S7 The effects of Nrf2 activation on DEHP-treated C2C12 myotubes. A-B. The mRNA expression of *Keap1*(A) and *Nrf2* (B) in DEHP-treated C2C12 myotubes (n = 3 independent experiments). *Gapdh* was used as the loading control. **C-G**. C2C12 myotubes were pretreated with 5 μM SFN before 25 μM DEHP exposure. **C**. The expression of *Nrf2* normalized by *Gapdh*. **D**. The content of GSH normalized to protein content. **E.** The expression of miR-200a normalized by U6. **F**. The expression of *Txnip* normalized by *Gapdh*. **G**. The Insulin-stimulated 2-DG uptake. All data were presented as the mean ± SEM. *P < 0.05, **P < 0.01 vs. corresponding control as indicated.

Figure S8

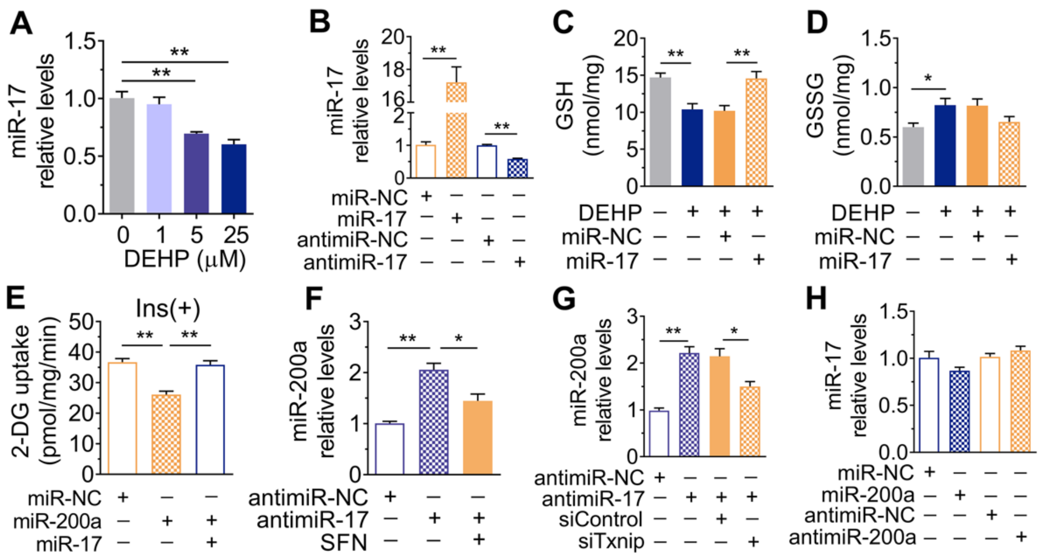

Figure S8 miR-17 negatively regulated oxidative stress and insulin signaling in C2C12 myotubes. A. The expression of miR-17 in C2C12 myotubes treated with serial concentrations of DEHP for 48 h (n = 3 independent experiments). U6 was used to normalized miR-17 expression. **B.** The expression of miR-17 in C2C12 myotubes transfected with 50 nM agomiR-17 or 200 nM antagomir-17 or corresponding controls for 48 h (n = 3 independent experiments). U6 was used to normalized miR-17 expression. **C-D**. The contents of GSH (C) and GSSG (D) normalized to protein content in C2C12 myotubes transfected with 50 nM agomiR-17 and treated with 25 μM DEHP (n = 3 independent experiments). The calculated GSH/GSSG ratio were shown in Figure 6O. **E**. The insulin-stimulated 2-DG uptake in C2C12 myotubes co-transfected with agomiR-200a and agomiR-17 or corresponding controls for 48 h (n = 3 independent experiments). **F**. The expression of miR-200a in C2C12 myotubes cotreated with antagomir-17 and SFN or corresponding control (n = 3 independent experiments). U6 was used to normalized miR-200a expression. **G**. The expression of miR-200a in C2C12 myotubes cotreated with antagomir-17 and si*Txnip* or corresponding control (n = 3 independent experiments). U6 was used to normalized miR-200a expression. **H.** The expression of miR-17 in C2C12 myotubes transfected with agomiR-200a, antagomir-200a or corresponding control for 48 h (n = 3 independent experiments). U6 was used to normalized miR-17 expression. All data were presented as the mean ± SEM. *P < 0.05, **P < 0.01 vs. corresponding control as indicated.

Figure S9

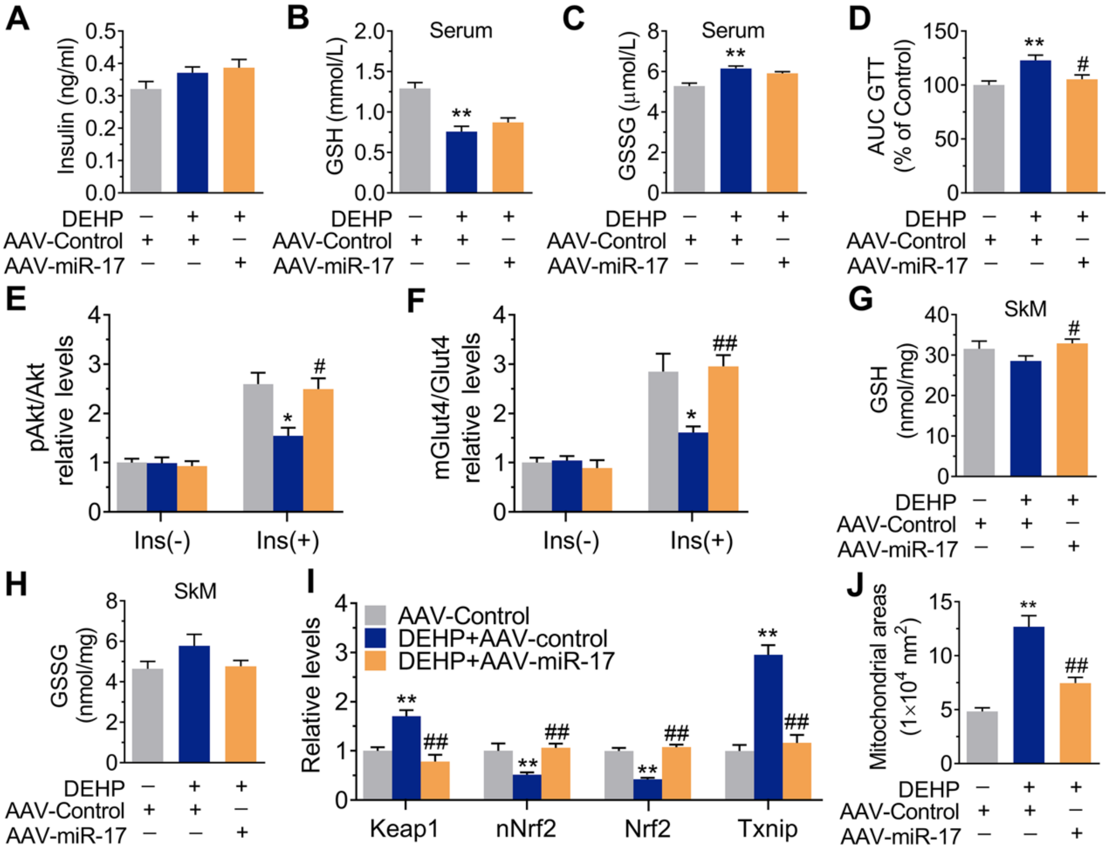

Figure S9 Overexpression of miR-17 in SkM was resistant to DEHP-induced oxidative stress and IR. A. The fasting serum insulin (n = 6 mice per group). **B-C.** The serum levels of GSH (B) and GSSG (C) (n = 5 mice per group). The calculated serum GSH/GSSG ratio were shown in Figure 7D. **D**. The AUC of the IPGTT in Figure 7F (n = 5 mice per group). **E-F**. The quantification data of pAkt (E) and mGlut4 (F) normalized by Gapdh. (n = 3 mice per group). The representative western blot images were shown in Figure 7I (n = 3 mice per group). **G-H**. The levels of GSH (G) and GSSG (H) normalized to protein content in SkM (n = 3 mice per group). The calculated GSH/GSSG ratio in SkM were shown in Figure 7K. **I**. The quantification data of protein expression of genes related to oxidative stress (n = 3 mice per group). The total protein was normalized by Gapdh and the protein expression of Nrf2 in nuclear were normalized by Lamin B1. The representative western blot images were shown in Figure 7M. **J**. The quantification of average mitochondrial area in SkM determining by manually circling 15 mitochondria per mice (n = 3 mice per group). The representative TEM images were shown in Figure 7O. All data were presented as the mean ± SEM. *P < 0.05 vs. control mice infected with AAV-Control, **P < 0.01 vs. control mice infected with AAV-Control. #P < 0.05 DEHP-exposed mice infected with AAV-Control vs. DEHP-exposed mice infected with AAV-miR-17, ##P < 0.01 DEHP-

exposed mice infected with AAV-Control vs. DEHP-exposed mice infected with AAV-miR-17.

Figure S10

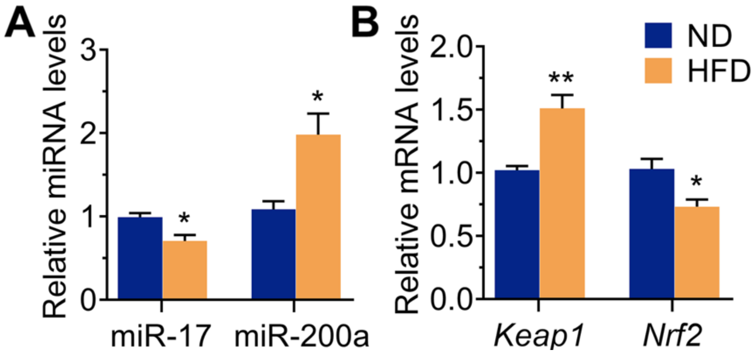

Figure S10 The mRNA expression of miR-17/Keap1-Nrf2/miR-200a cascade in SkM of mice fed a high-fat diet. Three-week-old male healthy C57BL/6 mice were fed with high-fat diet for 18 weeks (n = 4 mice per group). The high-fat diet (Xietong Organism Institute, Nanjing, China) contains 40.86% fat, 21.24% protein, and 37.9% carbohydrates, with energy of 4.398 kJ/g. *Gapdh* was used as the loading control. 18-week high-fat diet feeding induced similar changes in miR-17, Keap1, Nrf2 and miR-200a expression patterns in SkM of male C57BL/6 mice, compared with the mice model of DEHP-triggered IR. All data were presented as the mean ± SEM. *$P < 0.05$ mice fed with chow diet *vs.* mice fed with high-fat diet. **$P < 0.01$ mice fed with chow diet *vs.* mice fed with high-fat diet.

Tables

Table S1 Primers used in the construction of luciferase reporters.

Plasmid	Primer sequence (from 5’→3’)
p*Insr*-WT-luc	Forward:CCGCTCGAGAGATCATGGTTCTGGAACCC<br>Reverse:AATGCGGCCGCACTGTGCTTTGGATGGGTTT
p*Insr*-MT-luc	Forward:CATGTGTCATGTCACAGGGATCAAATGTGCCATA<br>Reverse: TTGATCCCTGTGACATGACACATGGGATATAACC
p*Irs1*-WT-luc	Forward: CCGCTCGAGTACTTCTGGGAAGGGTTGAG<br>Reverse: AATGCGGCCGCGCGAGTTCCTTGAAAACAAT
p*Irs1*-MT-luc	Forward: ACAATGTTGTCACAAGCAGATCTTAATCATTTGC<br>Reverse: AGATCTGCTTGTGACAACATTGTACATCTGGTTG
p*Keap*1-WT-luc	Forward: CCGCTCGAGCAACAAAACTGTACCTGCTG<br>Reverse: AATGCGGCCGCCATGGATTTGAGTTCTGGTC
p*Keap1*-MT-luc	Forward: GTACCTGCTCTTCGTGTTGGAATACCTGAGCA<br>Reverse: AGGTATTCCAACACGAAGAGCAGGTACAGTTT

Table S2 Primers used in qRT-PCR analyses of mRNAs.

Gene	Primer sequence (from 5’→3’)
Akt2	Forward: TTTGCACTCGAGAGATGTGG<br>Reverse: TTTGCACAAGCCAAAGTCAG
Dnmt1	Forward:CTTCACCTAGTTCCGTGGCTA<br>Reverse: CCCTCTTCCGACTCTTCCTT
Dnmt3a	Forward:GCACCAGGGAAAGATCATGT<br>Reverse: CAATGGAGAGGTCATTGCAG
Dnmt3b	Forward: GGATGTTCGAGAATGTTGTGG<br>Reverse: GTGAGCAGCAGACACCTTGA
Glut4	Forward:CATGGCTGTCGCTGGTTTC<br>Reverse: AAACCCATGCCGACAATGA
Gapdh	Forward: GCCAAGGTCATCCATGACAACT<br>Reverse: GAGGGGCCATCCACAGTCTT
Insr	Forward:AACAGATGCCACTAATCCTTC<br>Reverse: GCCCTTTGAGACAATAATCC
Irs1	Forward:CCAGCCTGGCTATTTAGCTG

	Reverse: CCCAACTCAACTCCACCACT
Irs2	Forward:TAGCCACAGGAGCAACACAC<br>Reverse: CAGGCGTGGTTAGGGAGTAA
Keap1	Forward: ATGAGCCAGAGCGGGACGAG<br>Reverse: GCATACAGCAAGCGGTTGAGC
Malat1	Forward: GGAGCCATACGGATGTGGTG<br>Reverse: GCGCAGTTGACAAGCCAAG
Nrf2	Forward: CTACTCCCAGGTTGCCCACA<br>Reverse: CGACTCATGGTCATCTACAAATGG
Pik3r1	Forward: GGAGGTGAAGCTGAGAGTGG<br>Reverse: TGTCCATCTGTCCTCCATCA
Pik3r5	Forward:GAGCCTACAGGAGCTGGTCA<br>Reverse: GGTGCCTTTCTCTTGGACCT
Txnip	Forward: TCAAGGGCCCCTGGGAACATC<br>Reverse: GACACTGGTGCCATTAAGTCAG

Akt2, thymoma viral proto-oncogene 2; *Dnmt1, DNA methyltransferase 1*; *Dnmt3a, DNA methyltransferase 3 alpha*; *Dnmt3b, DNA methyltransferase 3 beta*; *Glut4, solute carrier family 2 member 4*; *Gapdh, glyceraldehyde-3-phosphate dehydrogenase*; *Insr, insulin receptor*; *Irs1, insulin receptor substrate 1*; *Irs2, insulin receptor substrate 2*; *Keap1, kelch like ECH associated protein 1*; *Malat1, metastasis associated lung adenocarcinoma transcript 1*; *Nrf2, nuclear factor (erythroid derived 2) like 2*; *Pik3r1, phosphoinositide-3-kinase regulatory subunit 1*; *Pik3r5, phosphoinositide-3-kinase regulatory subunit 5*; *Txnip, thioredoxin interacting protein*.

Table S3 Primers used in qRT-PCR analyses of miRNAs.

RNA or miRNA	Genbank or miRBase seq#	Primer sequence (from 5'→3')
U6 RNA	NR_004394.1	Reverse transcription:<br>CGCTTCACGAATTTGCGTGTCAT<br>Forward: GCTTCGGCAGCACATATACTAAAAT<br>Reverse: CGCTTCACGAATTTGCGTGTCAT
miR-200a	MIMAT0000519	Reverse transcription:<br>gtcgtatccagtgcgtgtcgtggagtcggcaattgcactggatacgactACATCG<br>Forward: ggggTAACACTGTCTGGTAA<br>Reverse: tgcgtgtcgtggagtc
miR-141	MIMAT0000153	Reverse transcription:

		gtcgtatccagtgcgtgtcgtggagtcggcaattgcactggatacgactCCATCT<br>Forward: ggggTAACACTGTCTGGTAA<br>Reverse: tgcgtgtcgtggagtc
miR-200b	MIMAT0000233	Reverse transcription:<br>gtcgtatccagtgcgtgtcgtggagtcggcaattgcactggatacgactTCATCA<br>Forward: ggggTAATACTGCCTGGTAA<br>Reverse: tgcgtgtcgtggagtc
miR-200c	MIMAT0000657	Reverse transcription:<br>gtcgtatccagtgcgtgtcgtggagtcggcaattgcactggatacgactTCCATCA<br>Forward: ggggTAATACTGCCGGGTAA<br>Reverse: tgcgtgtcgtggagtc
miR-429	MIMAT0001537	Reverse transcription:<br>gtcgtatccagtgcgtgtcgtggagtcggcaattgcactggatacgactACGGCA<br>Forward: ggggTAATACTGTCTGGTAA<br>Reverse: tgcgtgtcgtggagtc
miR-17	MIMAT0000649	Reverse transcription:<br>gtcgtatccagtgcgtgtcgtggagtcggcaattgcactggatacgactCTACCT<br>Forward: ggggCAAAGTGCTTACAGTGC<br>Reverse: tgcgtgtcgtggagtc
